# Egr1 deficiency induces browning of inguinal subcutaneous white adipose tissue in mice

**DOI:** 10.1038/s41598-017-16543-7

**Published:** 2017-11-23

**Authors:** Cécile Milet, Marianne Bléher, Kassandra Allbright, Mickael Orgeur, Fanny Coulpier, Delphine Duprez, Emmanuelle Havis

**Affiliations:** 1 Sorbonne Universités, UPMC Univ Paris 06, CNRS UMR7622, Inserm U1156, IBPS-Developmental Biology Laboratory, F-75005 Paris, France; 2 University of Pittsburgh, Pittsburgh, Pennsylvania United States; 3 École normale supérieure, PSL Research University, CNRS, Inserm, Institut de Biologie de l’École normale supérieure (IBENS), Plateforme Génomique, 75005 Paris, France

## Abstract

Beige adipocyte differentiation within white adipose tissue, referred to as browning, is seen as a possible mechanism for increasing energy expenditure. The molecular regulation underlying the thermogenic browning process has not been entirely elucidated. Here, we identify the zinc finger transcription factor EGR1 as a negative regulator of the beige fat program. Loss of *Egr1* in mice promotes browning in the absence of external stimulation and leads to an increase of *Ucp1* expression, which encodes the key thermogenic mitochondrial uncoupling protein-1. Moreover, EGR1 is recruited to the proximal region of the *Ucp1* promoter in subcutaneous inguinal white adipose tissue. Transcriptomic analysis of subcutaneous inguinal white adipose tissue in the absence of *Egr1* identifies the molecular signature of white adipocyte browning downstream of *Egr1* deletion and highlights a concomitant increase of beige differentiation marker and a decrease in extracellular matrix gene expression. Conversely, *Egr1* overexpression in mesenchymal stem cells decreases beige adipocyte differentiation, while increasing extracellular matrix production. These results reveal a role for *Egr1* in blocking energy expenditure via direct *Ucp1* transcription repression and highlight *Egr1* as a therapeutic target for counteracting obesity.

## Introduction

White fat browning is a mechanism that produces heat and limits weight gain. The understanding of the molecular regulation underlying white fat browning has sparked interest to counteract obesity.

The adipose tissue of humans and other mammals contains white adipose tissue (WAT) and brown adipose tissue (BAT). WAT and BAT are developmentally and functionally distinct and contain white and brown adipocytes, respectively^1–3^. More recently, a third type of adipocytes has been described within WAT, beige adipocytes. Morphological and molecular analyses showed that brown and beige adipocytes are remarkably similar and express the same thermogenic markers^4^. However beige adipocytes, in contrast to brown adipocytes, express thermogenic markers only after external stimulations, such as cold exposure, starvation, exercise or hormone treatment^5^. In the adult, beige adipocytes are produced by the trans-differentiation of mature white adipocytes^4^ or by *de novo* differentiation of progenitors^6^ in response to external stimulations. This process is referred to as “browning” or “beigeing”^2,7^.

Because the increase of WAT is observed in many metabolic diseases, WAT browning represents a promising therapeutic approach. Consequently, it is crucial to decipher the molecular aspects underlying the beige differentiation program. Adipogenesis is triggered by a core adipogenic network, starting with the expression of *Cebpb* (CCAAT/enhancer binding protein ß), which activates the expression of *Pparg* (Peroxisome proliferator-activated receptor γ) and *Cebpa* (CCAAT/enhancer binding protein α), which in turn activates *Ppara* (Peroxisome proliferator-activated receptor α) expression^8^. Consistent with its thermogenic function, brown/beige differentiated adipocytes express high levels of UCP1, a mitochondrial protein that uncouples oxidative phosphorylation from ATP synthesis^9,10^. The Krebs cycle enzymes, such as OGDH (oxoglutarate dehydrogenase), SUCLA2 (succinate-Coenzyme A ligase) and COX8B (Cytochrome C Oxidase Subunit VIIIb)^11,12^ are also involved in heat production in beige/brown adipose tissue. Consistent with their anti-fat function, brown/beige differentiated adipocytes express factors involved in lipolysis such as PLIN5 (Perilipin 5^13^) and CIDEA (Cell Death-Inducing DFFA-Like Effector A^12^). Beige adipocyte differentiation relies on the expression of a set of transcriptional activators^2,3^. PRDM16 (PR domain containing 16) is considered as a master regulator of the brown/beige program via direct interaction with transcription factors, such as C/EBPβ, PPARα, PPARγ, and PGC-1α (Peroxisome proliferator-activated receptor Gamma Coactivator 1-alpha^14–16^). Of note, beige and white differentiation programs share transcriptional regulators, such as C/EBPβ, which has been shown to be sufficient for *Ucp1* transcription via direct binding to the *Ucp1* proximal promoter *in vitro* ^17,18^. Moreover, *Cebpb* mutant mice display defective thermoregulation^19^. In addition to transcriptional regulators, growth factors such as FGF21 (Fibroblast Growth Factor-21) and BMP4 (Bone morphogenetic Protein-4), adipokines such as leptin and hormones such as T_3_ (Triiodothyronin 3) have been identified as being able to induce the brown/beige fat phenotype^2,20,21^. The T_4_ to T_3_ converting enzyme Desiodase 2 (DIO2) is also involved in the browning process^22^.

The zinc finger transcription factor EGR1 (Early Growth Response-1) is involved in multiple processes including cell proliferation, differentiation, migration, apoptosis, and inflammation in many cell types^23–27^. *Egr1* is expressed in adult adipose tissues^28,29^ where its overexpression has been linked to obesity and obesity-associated metabolic disorders in both humans and mouse models^28,29^. Conversely, Egr1-deficient mice are protected from diet-induced obesity^29^. Consistently, EGR1 inhibits lipolysis and promotes fat accumulation in cultured adipocytes by directly repressing the transcription of the adipose triglyceride lipase (ATGL) gene^30^. Surprisingly, *Egr1* overexpression represses white adipocyte differentiation in the 3T3-L1 and C3H101/2 cell lines^31,32^.

To understand how *Egr1* can both be linked with obesity and adverse metabolic outcomes while repressing differentiation of white adipocytes in culture, we investigated the role of *Egr1* in white adipose tissue development during the postnatal period in female mice. We analysed the consequences of *Egr1* inhibition for subcutaneous inguinal white adipose tissue (SC-WAT) formation during postnatal and adult periods, using a mouse model deficient for *Egr1*, with no external stimulation. We also assessed the consequences of *Egr1* overexpression for beige differentiation in mesenchymal stem cells.

## Results and Discussion

### *Egr1*^−/−^ mice display inguinal subcutaneous white adipose tissue browning with no external stimulation

**Figure 1:**
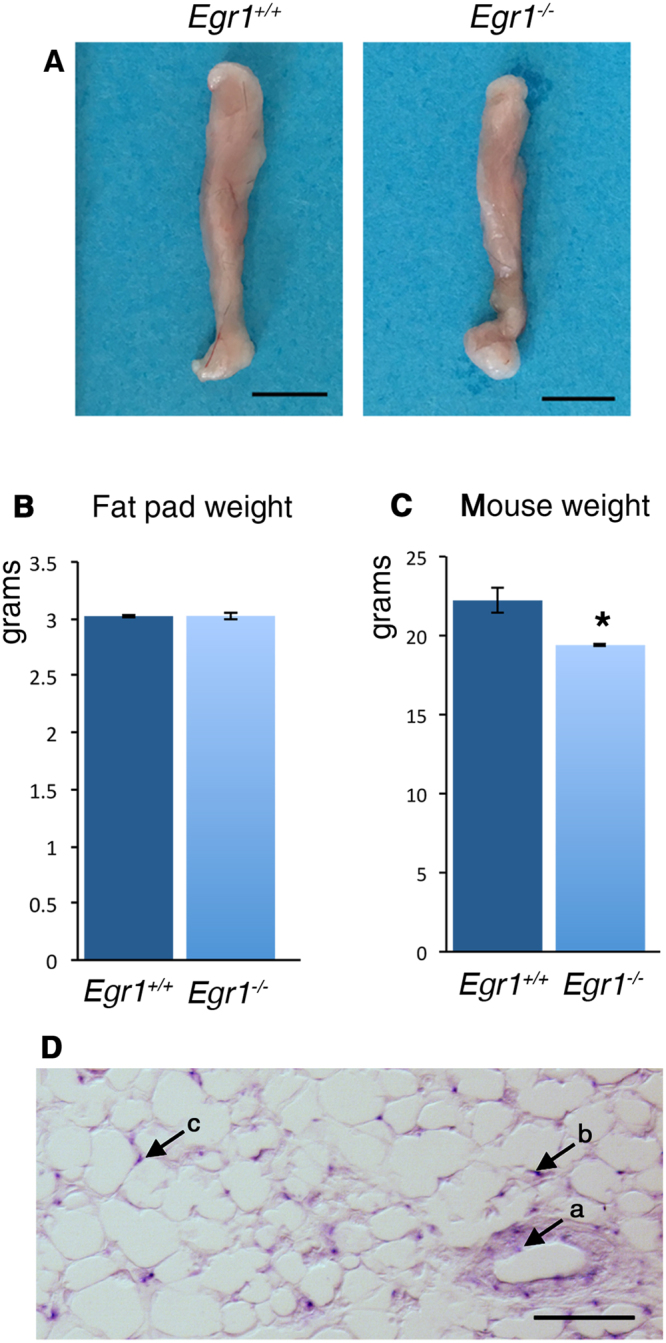
Phenotype of inguinal subcutaneous white adipose tissue in 4-month-old *Egr1* ^−/−^ mice. (**A**) Pictures of fat pads (SC-WAT) from 4-month-old *Egr1* ^+/+^ and *Egr1* ^−/−^ mice. Scale bars: 5 mm. (**B**) Weight in grams of SC-WAT of 4-month-old *Egr1* ^+/+^ and *Egr1* ^−/−^ mice. The graph shows mean ± standard deviations of 6 *Egr1* ^+/+^ fat pads and 8 *Egr1* ^−/−^ fat pads. (**C**) Weight in grams of 4-month-old wild-type and mutant mice. The graph shows means ± standard deviations of 4 *Egr1* ^+/+^ and 4 *Egr1* ^−/−^ mice. The p-value was obtained using the Mann-Whitney test. Asterisk indicates the p-value *P < 0.05. (**D**) SC-WAT of 1-month-old mice was longitudinally sectioned. 6 µm sections were hybridized with the DIG-labeled antisense probe for *Egr1* (blue). Arrow a points *Egr1* expression in blood vessels. Arrows b and c indicate *Egr1* expression in white adipocytes. Scale bars: 50 µm.

The subcutaneous inguinal white adipose tissue (SC-WAT) expands during the post-natal period^33^ and is the largest white fat depot in mice^10,34^. Moreover, mouse SC-WAT is comparable in terms of location to the large gluteofemoral subcutaneous depot in humans that is linked to increased risk of developing obesity-related morbidities and mortality^35^. The size and weight of SC-WAT fat pads were similar in *Egr1* ^*+/+*^ and *Egr1* ^−/−^ 4-month-old mice, although the total body weight was slightly reduced in *Egr1* ^−/−^ mice compared to control mice (Fig. 1A–C). These observations suggest that Egr1 loss-of-function leads to a reduced body weight via an increased numbers of active beige adipocytes and potentially through unknown global metabolic processes. *Egr1* expression in SC-WAT was detected in blood vessels (Fig. 1D, arrow a) as previously described^36^ and in adipocytes (Fig. 1D, arrows b,c).

**Figure 2:**
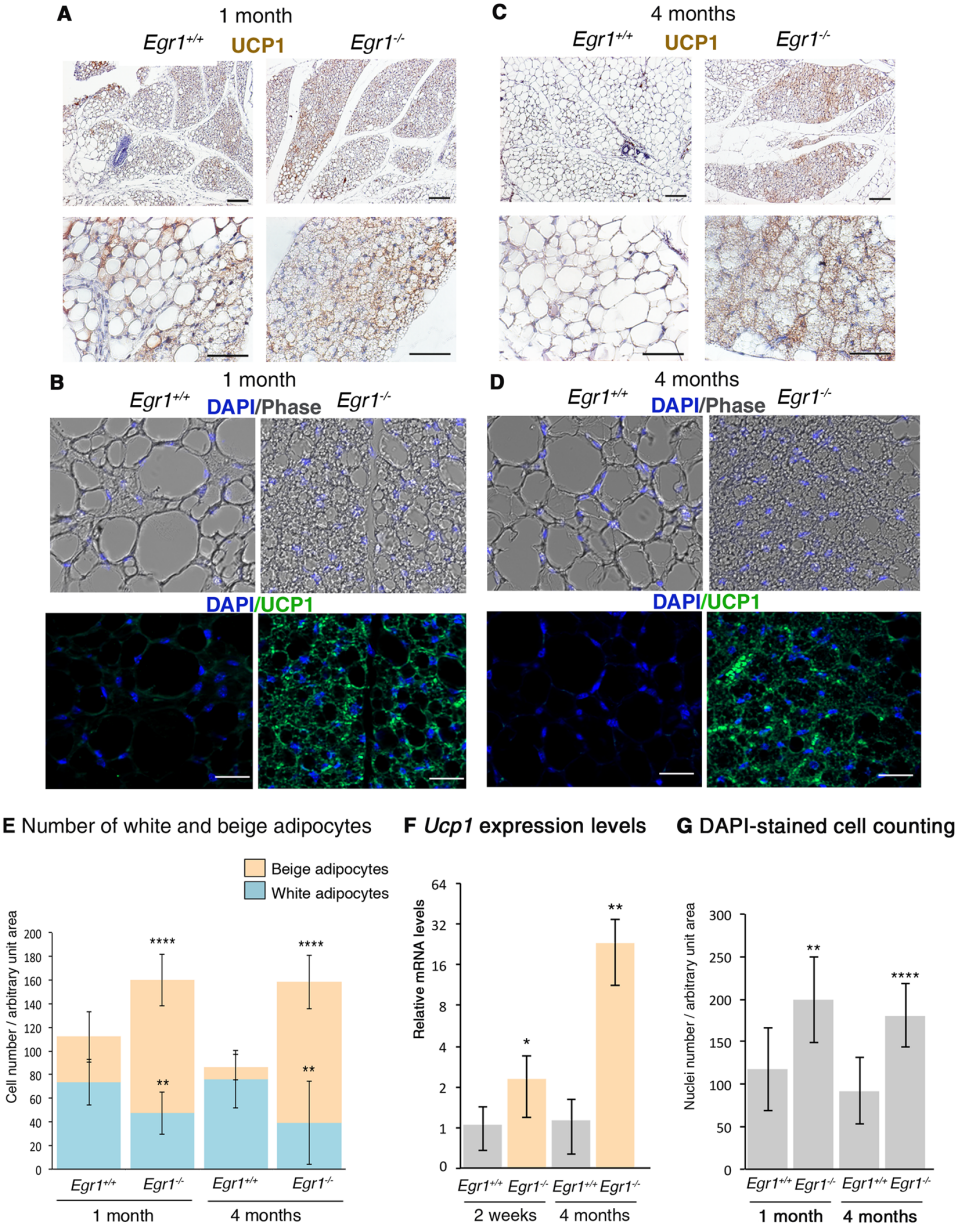
*Egr1* leads to inguinal subcutaneous white adipose tissue browning in postnatal and 4 month-old mice. (**A**–**D**) Sections of SC-WAT of 1-month-old (**A**,**B**) and 4-month-old (**C**,**D**) *Egr1* ^+/+^ and *Egr1* ^−/−^ mice were immuno-stained with UCP1 antibody. Nuclei were visualized with hematoxilin (**A**,**C**) or DAPI (**B**,**D**). (**B**,**D**) upper and lower panels are Dapi/Phase and UCP1/DAPI views of the same field. Scale bars: (**A**,**C**) lower magnification 100 µm, higher magnification 50 µm; (**B**,**D**) 25 µm. (**E**) White and beige adipocyte number was counted in arbitrary unit areas of transverse sections of SC-WAT of 1-month-old Egr1^+/+^ (N = 10) and *Egr1* ^−/−^ (N = 11) mice and 4-month-old *Egr1* ^+/+^ (N = 13) and *Egr1* ^−/−^ (N = 14) mice. Graphs show means of counts generated from 10 to 14 sections for each sample ± standard deviations. Asterisks indicate the p-values obtained using the Mann-Whitney test, comparing beige or white adipocyte number between mutant and control mice **P < 0.01, ****P < 0.0001. (**F**) RT-qPCR analysis of expression levels for beige adipocyte differentiation marker *Ucp1* in SC-WAT of 2-week-old and 4-month-old *Egr1* ^−/−^ mice compared to *Egr1* ^+/+^ mice. Graphs show means ± standard deviations of 5 samples from 2-week-old *Egr1* ^+/+^ and *Egr1* ^−/−^ mice, 6 samples from 4-month-old wild-type mice and 5 samples from 4-month-old *Egr1* ^−/−^ mice. The *Ucp1* mRNA levels of control (*Egr1* ^+/+^) SC-WAT were normalized to 1. The relative mRNA levels were calculated using the 2^−ΔΔCt^ method. The p-values were obtained using the Mann-Withney test. Asterisks indicate the p-values *p < 0.05, **p < 0.01. (**G**) Cell number in SC-WAT in *Egr1* ^+/+^ and *Egr1* ^−/−^ mice. Number of nuclei (DAPI-positive cells) was counted in arbitrary unit areas of transverse sections of SC-WAT of 1 month-old *Egr1* ^+/+^ (N = 10) and *Egr1* ^−/−^ (N = 11) mice and 4 month-old *Egr1* ^+/+^ (N = 13) and *Egr1* ^−/−^ (N = 11) mice. Graphs show means of 10 to 13 sections for each sample ± standard deviations. Asterisks indicate the p-values obtained using the Mann-Whitney test, comparing beige or white adipocyte number between mutant and control mice **P < 0.01, **** P < 0.0001.

The beige adipocytes in SC-WAT of *Egr1* ^+/+^ and *Egr1* ^−/−^ mice were identified by the expression of UCP1 detected both by immunodetection with DAB staining (Fig. 2A,C) or fluorescent staining (Fig. 2B,D) and by the multilocular aspect of the lipid droplets observed in phase contrast images. The white adipocytes were identified by a unilocular lipid droplet observed in phase contrast images (Fig. 2A–D). SC-WAT from 1-month-old (post-natal) and 4-month-old (adult) *Egr1* ^−/−^ mice exhibited increased UCP1 staining compared to *Egr1* ^+/+^ mice (Fig. 2A–D). At birth and during post-natal period, the inguinal subcutaneous adipose tissue of wild-type mice contains beige adipocytes that are out-numbered by the development of increasing numbers of white adipocytes^37^ (Fig. 2E). In contrast, we observed a significant increase of the proportion of beige adipocytes and a reduction in the proportion of white adipocytes in SC-WAT of *Egr1* ^−/−^ mice compared to *Egr1* ^+/+^ mice (Fig. 2E). Consistently, the *Ucp1* mRNA expression levels were increased in *Egr1*-deficient SC-WATs compared to equivalent control SC-WATs (Fig. 2F). SC-WATs of *Egr1* ^−/−^ mice exhibited a higher number of cells compared to *Egr1* ^+/+^ mice at 1 and 4 months of age (Fig. 2G), suggesting that EGR1 repressed adipocyte proliferation. However, we did not observe any difference in cell proliferation rates between 1-month-old and 4-month-old *Egr1* ^−/−^ mice compared to respective controls (see Supplementary Fig. 1). These observations suggest that EGR1 affects cell proliferation of adipocyte progenitors at early stages of adipose tissue development, before 1 month of age. Contrary to our observations, EGR1 has recently been identified to promote self-renewal of adipose progenitors of chin and knee in physio-pathological conditions^38^. These opposite results highlight the heterogeneity of adipocyte progenitors and adipocytes depending on the fat pad location. Heterozygous *Egr1* ^+/−^ mice exhibited an intermediate browning phenotype compared to *Egr1* ^+/+^ and *Egr1* ^−/−^ 4-month-old mice (see Supplementary Fig. 2A). We observed an increase in the number of adipocytes and a higher proportion of beige adipocytes in SC-WAT of *Egr1* ^+/−^ mice compared to *Egr1* ^+/+^ mice (see Supplementary Fig. 2B–C). However, the increase was smaller compared to *Egr1* ^−/−^ mice (Fig. 1E,G).

We conclude that the increase of *Ucp1* transcript levels, of UCP1 protein and in the density of UCP1+ cells in SC-WAT of *Egr1* ^−/−^ mice (Fig. 2) evidences an increase of WAT browning in *Egr1* ^−/−^ mice with no external stimulation. This result is consistent with the UCP1 increase in visceral perigonadal white adipose previously observed in *Egr1* ^−/−^ mice under high fat diet feeding^29^. We conclude that *Egr1* deficiency promotes spontaneous SC-WAT browning without external stimulation. These results indicate that the presence of *Egr1* in white adipocytes represses spontaneous WAT browning.

### Molecular signature of inguinal subcutaneous white adipose tissue browning downstream of *Egr1*

**Figure 3:**
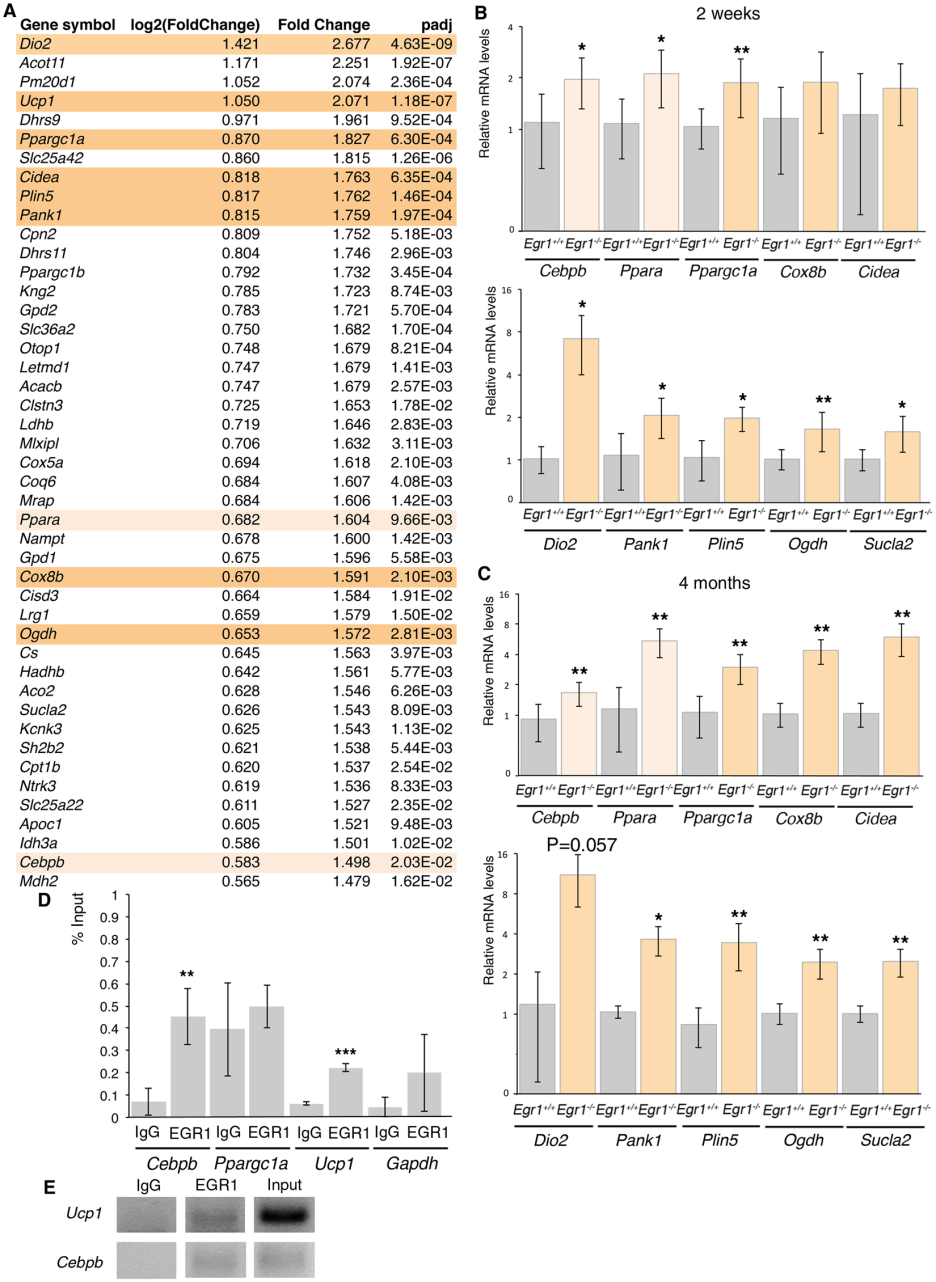
Transcriptomic analysis of subcutaneous inguinal adipose tissue of postnatal *Egr1* ^−/−^ versus *Egr1* ^+/+^ mice shows upregulation of beige adipocyte markers. (**A**) List of the first 45 upregulated genes in 6 inguinal subcutaneous fat pads of 3 *Egr1* ^−/−^ versus 3 *Egr1* ^+/+^ 2-week-old mice. (**B**,**C**) RT-qPCR analysis of the expression levels for generic adipocyte differentiation markers *Cebpb*, *Ppara*, beige adipocyte differentiation marker, *Ppargc1a*, *Cox8b*, *Cidea*, *Dio2*, *Pank1*, *Plin5*, *Ogdh* and *Sucla2* in SC-WAT of 2-week-old (**B**) and 4-month-old (**C**) *Egr1* ^−/−^ mice compared to *Egr1* ^+/+^ mice. For each gene, the mRNA levels of control (*Egr1* ^+/+^) SC-WAT were normalized to 1. Graphs show means ± standard deviations of 5 samples from 2-week-old *Egr1* ^+/+^ mice and *Egr1* ^−/−^ mice, 6 samples from 4-month-old *Egr1* ^+/+^ mice and 5 samples from *Egr1* ^−/−^ mice. The relative mRNA levels were calculated using the 2^−ΔΔCt^ method. The p-values were obtained using the Mann-Withney test. Asterisks indicate the p-values *p < 0.05, **p < 0.01. (**D**) Chromatin Immunoprecipitation (ChIP) assays were performed from 60 fat pads of wild type 2-week-old mice with antibodies against EGR1 or IgG2 as irrelevant antibody in three independent biological experiments. ChIP products were analyzed by RT-q-PCR (N = 2). Primers targeting the proximal promoter regions of *Cebpb* and *Ucp1* revealed the recruitment of EGR1 in the vicinity of these sequences, while primers targeting the proximal promoter regions of *Ppargc1a* and *Gapdh* (negative controls) did not show any immunoprecipitation with EGR1 antibody compared to IgG2 antibody. Results were represented as percentage of the input. Error bars showed standard deviations. The p-values were obtained using the Mann-Withney test. Asterisks indicate the p-values, **p < 0.01, ***p < 0.001. (**E**) ChIP-qPCR samples were loaded on agarose gel and confirmed a specific amplification of *Cebpb* and *Ucp1* promoter regions after chromatin immunoprecipitation using EGR1 antibody. No DNA was immunoprecipitated by irrelevant IgG antibody. Input chromatin was diluted 2 times for *Ucp1* qPCR and 4 times for *Cebpb* qPCR.

In order to define the molecular signature underlying WAT browning downstream of Egr1, we performed RNA-sequencing of SC-WAT of 2-week-old *Egr1* ^+/+^ and *Egr1* ^−/−^ mice. 336 differentially expressed genes were significantly detected in *Egr1*-deficient SC-WAT compared to control SC-WAT. The 132 upregulated differentially expressed genes (Fig. 3A, see Supplementary Fig. 3) were subjected to functional annotation clustering according to their Gene Ontology (GO) classification, in the “*Biological Process*” category (see Supplementary Fig. 4). Among the 132 upregulated genes, the GO terms “*NADH metabolic process*”, “*Tricarboxylic acid cycle*”, “*Brown fat cell differentiation*” and “*Fatty acid metabolic process*” exhibited the highest enrichment scores (see Supplementary Fig. 4). Consistent with the beige phenotype (Fig. 2), the key beige adipocyte markers, *Ppargc1a*, *Ucp1*, *Cox8b*, *Cidea* ^7^ and other genes known to be involved in the beige differentiation program, *Dio2*, *Pank1*, *Plin5*, *Ogdh* and *Sucla2* ^11,22,39,40^ were identified as upregulated genes (Fig. 3A). The increased expression of these beige genes was confirmed by RT-qPCR at 2 weeks and 4 months of age (Fig. 3B,C). In addition, the generic adipogenesis regulators also known to be involved in beige differentiation, *Cepbb* ^41^ and *Ppara* ^42^ displayed an increased expression in *Egr1*-deficient SC-WAT (Fig. 3A–C). Interestingly, there was no modification of expression of signalling molecules controlling beige differentiation such as FGF21, BMP4 or Leptin. This indicates that the transcription factor EGR1 negatively regulates the transcription of beige differentiation markers. To test whether this regulation was direct, we performed Chromatin immunoprecipitation (ChIP) experiments from the SC-WAT of 2-week-old mice on key beige markers. EGR1 was recruited to the *Ucp1* proximal promoter in SC-WAT (Fig. 3D,E), showing a direct transcriptional regulation by EGR1. EGR1 was also recruited to the *Cebpb* promoter (Fig. 3D,E) but not to that of *Ppapgc1* gene (Fig. 3D), highlighting a direct and an indirect transcriptional regulation of these two genes by EGR1. These results show that EGR1 exerts its transcriptional repression of the beige program at two levels at least, through the direct recruitment of the main beige differentiation marker *Ucp1* and also through the direct recruitment to the *Cebpb* gene, which is known to regulate *Ucp1* transcription^17^.

**Figure 4:**
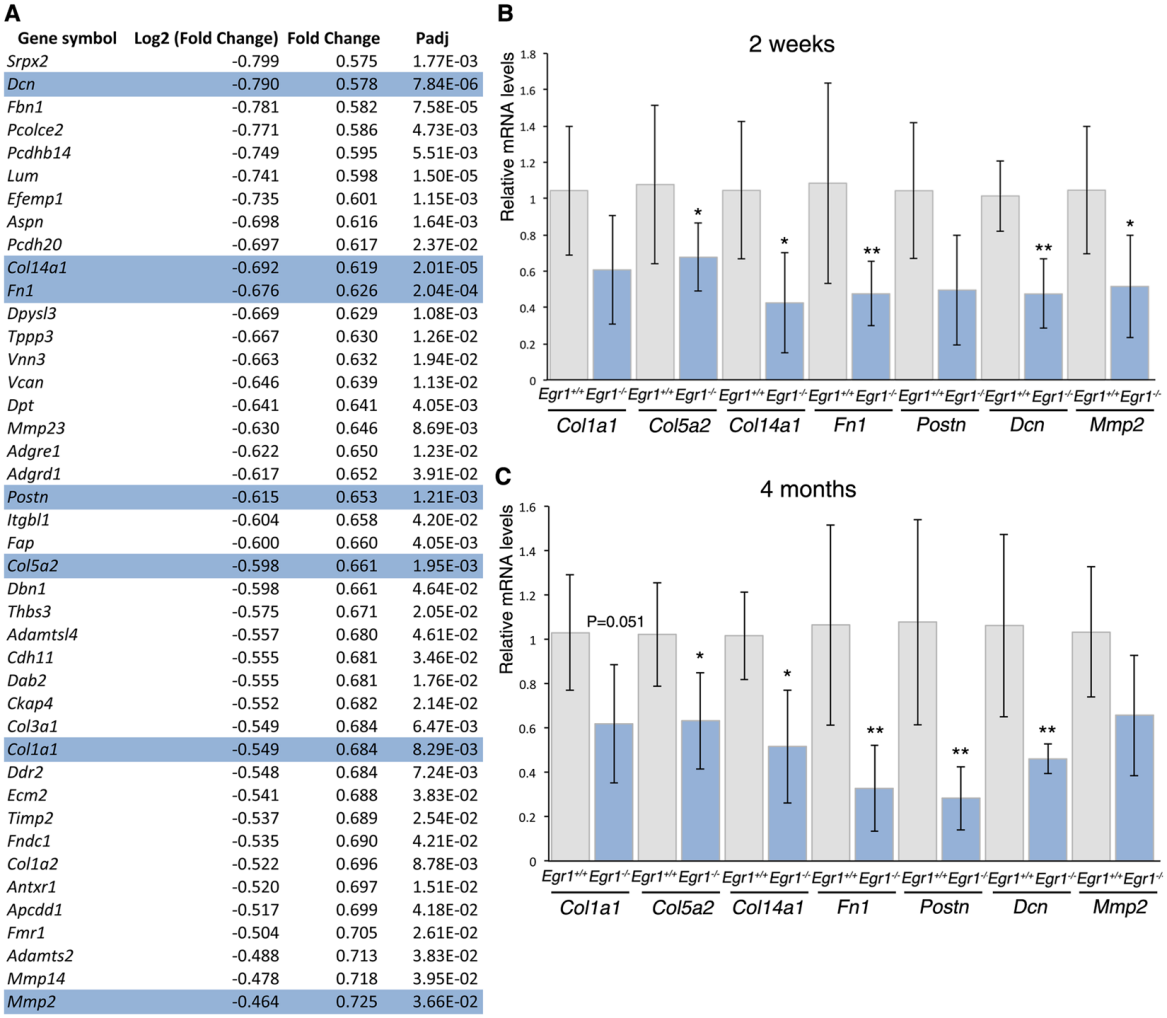
Transcriptomic analysis of the subcutaneous inguinal adipose tissue of postnatal *Egr1* ^−/−^ versus *Egr1* ^+/+^ mice reveals downregulation of extracellular matrix genes. (**A**) List of downregulated extracellular matrix genes in 6 inguinal subcutaneous fat pads of 3 *Egr1* ^−/−^ versus 3 *Egr1* ^+/+^ 2-week-old mice. (**B**,**C**) RT-qPCR analysis of gene expression levels for extracellular matrix genes, *Col1a1*, *Col5a2*, *Col14a1*, *Fn1*, *Postn*, *Dcn* and *Mmp2*, in SC-WAT of 2-week-old (**B**) and 4-month-old (**C**) *Egr1* ^+/+^ and *Egr1* ^−/−^ mice. For each gene, the mRNA levels of control (*Egr1* ^+/+^) SC-WAT were normalized to 1. Graphs show means ± standard deviations of 5 samples from 2-week-old *Egr1* ^+/+^ mice and *Egr1* ^−/−^ mice, 6 samples from 4-month-old *Egr1* ^+/+^ mice and 5 samples from *Egr1* ^−/−^ mice. The relative mRNA levels were calculated using the 2^−ΔΔCt^ method. The p-values were obtained using the Mann-Withney test. Asterisks indicate the p-values *p < 0.05, **p < 0.01.

The 204 downregulated differentially expressed genes (Fig. 4A, see Supplementary Fig. 5) in SC-WAT of *Egr1* ^−/−^ mice were enriched for the GO terms *“Collagen fibril organization*”, “*Collagen catabolic process*” and “*Extracellular matrix organization*” (see Supplementary Fig. 6). WAT produces extracellular matrix (ECM) whose composition and remodelling is crucial for adipocyte function^43^. Conversely, the expansion of adipose tissue during obesity leads to tissue remodelling and is associated with overexpression of *Col1a1*, *Col5a2*, *Fn1*, *Dcn* and the matrix metalloprotease *Mmp2* genes^44–48^. In the transcriptome of *Egr1*-deficient SC-WAT, *Col1a1*, *Col1a2*, *Col5a2*, *Col14a1*, *Fn1*, *Post*, *Dcn* and *Mmp2* were downregulated (Fig. 4A), which was confirmed by RT-qPCR in SC-WAT of 2 week- and 4 month-old mice (Fig. 4B,C). We conclude that *Egr1*-deficiency represses ECM genes associated with obesity. Our results indicate that WAT browning is associated with alteration of ECM composition. The inverse correlation between WAT browning and ECM is consistent with the suppression of brown adipogenesis in favour of fibrogenesis in mice^49^.

The concomitant upregulation of beige differentiation genes and downregulation of ECM genes is a signature of WAT browning downstream of *Egr1* deletion without any external stimulation.

### Mutual exclusive expression of Fibronectin and UCP1 in inguinal subcutaneous adipose tissue of *Egr1*^−/−^ mice

**Figure 5:**
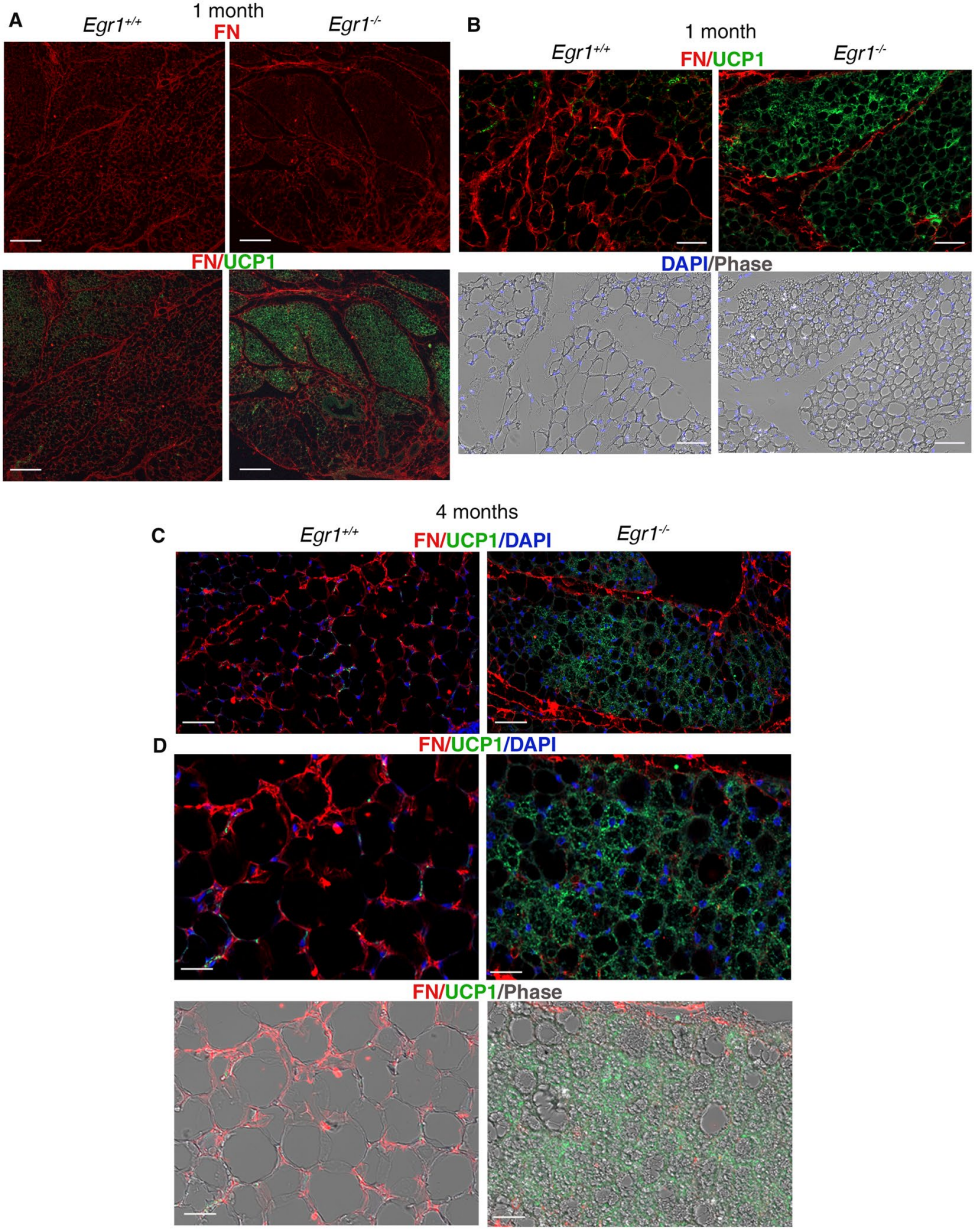
Fibronectin localization in SC-WATs of *Egr1* ^+/+^ and *Egr1* ^−/−^ mice. SC-WATs of 1-month-old (**A**,**B**) and 4-months-old (**C**,**D**) *Egr1* ^+/+^ and *Egr1* ^−/−^ mice were sectioned transversely and immuno-stained with Fibronectin FN (red) and UCP1 (green) antibodies. Nuclei were visualized with DAPI (blue). Individual channel or merged channels are indicated in panels. (**A**) Low magnifications show that FN (red) is less expressed in UCP1-positive areas (green) compared to UCP1-negative areas in 1-month-old *Egr1* ^+/+^ and *Egr1* ^−/−^ mice. Scale bars, 200 µm. (**B**) High magnifications show that FN is absent around UCP1 + beige adipoctytes in 1-month-old *Egr1* ^−/−^ compared to *Egr1* ^+/+^ mice, while being present around white adipoctyes in *Egr1* ^+/+^ mice. Scale bars, 50 µm. (**C**,**D**) At 4 months of age, FN is also absent around UCP1 + beige adipoctyes of SC-WATs from *Egr1* ^−/−^, while being present around white adipocytes in *Egr1* ^+/+^ mice. Scale bars, (**C**) 50 µm (**D**) 25 µm. (**B**–**D**) FN surrounds the regions of beige adipocytes.

Adipocyte ECM is an important component of white adipogenesis in normal or pathological conditions^43,44,48^. However, little is known for ECM-adipocyte interaction during beige adipogenesis. The ECM protein Fibronectin (FN) has been linked with obesity and adipose tissue fibrosis^50^. EGR1 has been previously shown to directly regulate *Fn1* transcription in human glioblastoma cells^51^. We analysed FN expression in SC-WAT in Egr1 deletion conditions (Fig. 5). Although immunohistochemistry on tissue section is not a quantitative technique, we observed a global decrease of FN expression in SC-WAT, concomitant with an increase of UCP1 expression in 1 month *Egr1* ^−/−^ compared to *Egr1* ^+/+^ mice (Fig. 5A). The decrease of FN protein (Fig. 5A) was fully consistent with the down-regulation of *Fn1* mRNA levels (Fig. 4B,C) in *Egr1*-deficient SC-WAT. FN expression was not observed around the UCP1 + beige adipocytes in SC-WAT of 1-month-old *Egr1* ^−/−^ mice, while FN was produced by white adipocytes in 1-month-old *Egr1* ^+/+^ mice (Fig. 5B). It has to be noticed that FN was observed surrounding the beige adipocyte areas (Fig. 5B). Similar results were found at 4 months of age, where FN was not detected around UCP1+ beige adipocytes, while being expressed around white adipocytes in 4-month-old *Egr1* ^+/+^ mice and *Egr1* ^+/+^ mice, respectively (Fig. 5D). We conclude that WAT browning in *Egr1* ^−/−^ mice is associated with an absence of FN expression by UCP1+ beige adipocytes. This result highlights an inverse correlation between the browning process and the expression of the ECM protein FN.

### Forced *Egr1* expression in mouse mesenchymal stem cells reduces beige marker expression and promotes extracellular matrix gene expression

**Figure 6:**
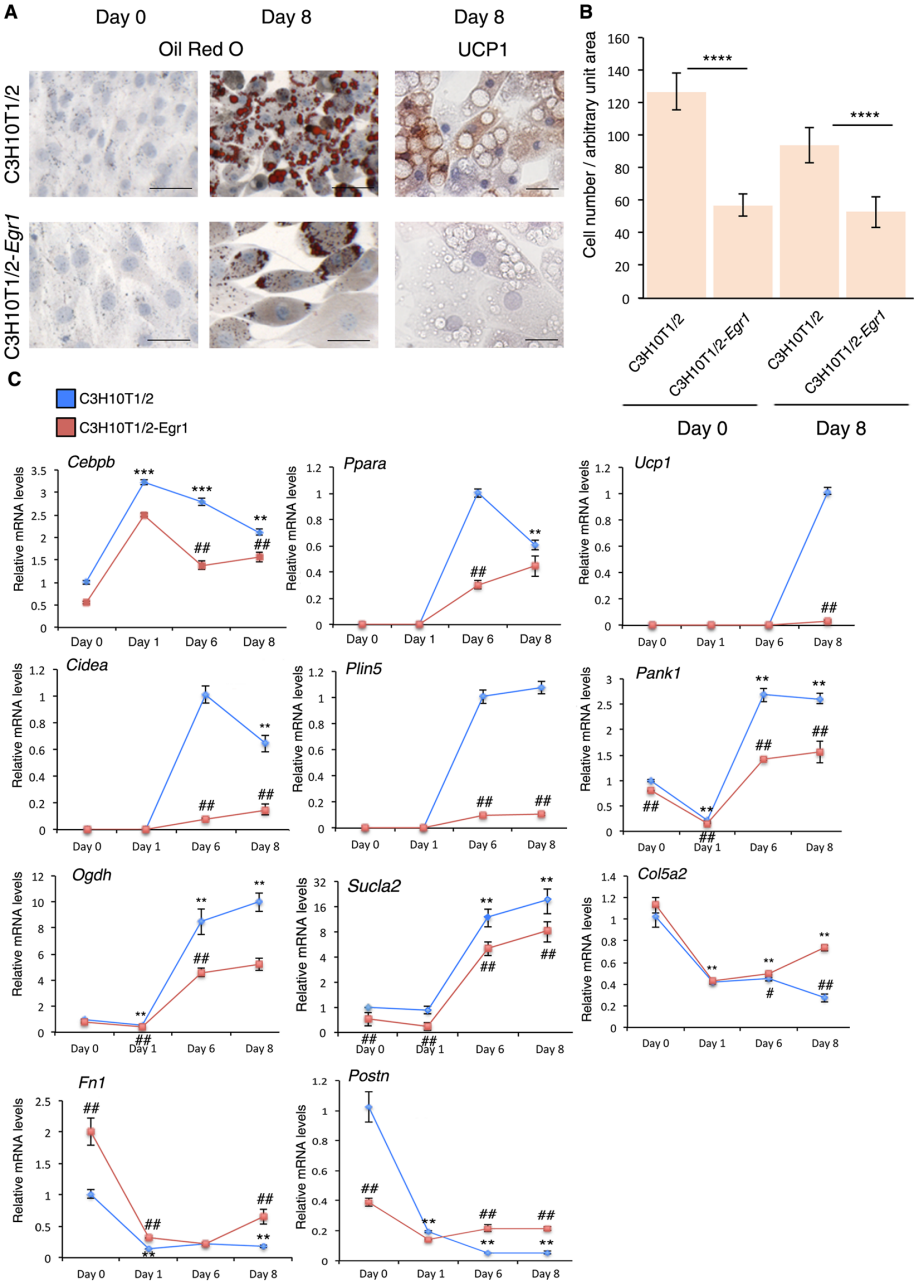
*Egr1* gain-of-function decreases beige adipocyte differentiation in mouse mesenchymal stem cells. (**A**) C3H10T1/2 and C3H10T1/2-*Egr1* cells subjected to beige adipocyte differentiation for 8 days were then stained with Oil Red O and Hematoxilin/Eosin at Day 0 (confluence) and Day 8, or immuno-stained with UCP1 antibody and counterstained with Hematoxilin/Eosin at Day 8. Scale bars: Oil red O staining 50 µm, UCP1 immunostaining 25 µm. (**B**) C3H10T1/2 and C3H10T1/2-Egr1 cell density at day 0 and after 8 days in beige differentiation medium. Graphs show means ± standard deviations of cell number from 10 pictures in each condition. The p-values were obtained using the Mann-Whitney test. Asterisks indicate the p-value ****P < 0.0001. (**C**) RT-qPCR analysis of the expression levels for the adipocyte transcriptional activators *Cebpb* and *Ppara*, the beige markers, *Ucp1*, *Cidea*, *Plin5*, *Pank1*, *Ogdh*, *Sucla2* and the extracellular components *Col5a2*, *Fn1* and *Postn* in C3H10T1/2 and C3H10T1/2-*Egr1* cells subjected to beige adipocyte differentiation. For each gene, the mRNA levels of the control C3H10T1/2 cells at Day 0 or from the first day of detection were normalised to 1. *Cebpb*, *Pank1*, *Ogdh*, *Sucla2*, *Col5a2*, *Fn1 and Postn* expression was detected from Day 0, *Ppara*, *Cidea* and *Plin5* expression was detected from Day 6, *Ucp1* expression was detected at day 8. The graphs show the relative levels of mRNAs in C3H10T1/2 and C3H10T1/2-*Egr1* cells at different time points (Day 0, Day 1, Day 6, and Day 8) of beige adipocyte differentiation compared to C3H10T1/2 cells at Day 0 or to C3H10T1/2 cells from the first day of gene detection. For each time point, graphs show means ± standard deviations of 6 samples. The p values were calculated using the Mann-Withney test. The relative mRNA levels were calculated using the 2^^−ΔΔCt^ method. Asterisks indicate the p-values of gene expression levels in C3H10T1/2-*Egr1* cells or C3H10T1/2 cells compared to Day 0 (*Ogdh* and *Col5a2*) or from the first day of gene detection (*Ppara*, *Cidea*, *Ucp1* and *Plin5*), **P < 0.01. ^#^Indicate the p-values of gene expression levels in C3H10T1/2-*Egr1* versus C3H10T1/2 cells, for each time point, ^#^P < 0.05, ^##^P < 0.01.

The spontaneous WAT browning in *Egr1* ^−/−^ mice and the direct transcriptional regulation of *Ucp1* gene by EGR1 in SC-WAT suggested that EGR1 repressed beige adipocyte differentiation. EGR1 gain-of-function experiments were performed in mouse mesenchymal stem cells, C3H10T1/2 cells, cultured under beige adipocyte differentiation conditions. Consistent with the increase in the number of adipocytes in SC-WAT of *Egr1* ^−/−^ (Fig. 2G), we observed a decreased number of C3H10T1/2-*Egr1* cells compared to C3H10T1/2 cells at day 0 and after 8 days of culture in the beige differentiation medium (Fig. 6A,B). Under beige stimulation, C3H10T1/2 cells acquired a beige phenotype, visualized by the appearance of numerous small lipid droplets and UCP1 expression within their cytoplasm (Fig. 6A). In contrast, C3H10T1/2-*Egr1* cells did not express UCP1 under beige stimulation, showing that EGR1 repressed the expression of the key thermogenic beige marker (Fig. 6A). Consistent with the absence of UCP1 protein (Fig. 6A), *Ucp1* mRNA levels were never increased in the context of *Egr1* overexpression (Fig. 6C). This observation fits with EGR1 recruitment to *Ucp1* promoter observed in SC-WAT (Fig. 3D,E). However, small lipid droplets were still observed in C3H10T1/2-*Egr1* cells, indicating that EGR1 repressed part of the beige phenotype through the repression of UCP1, but did not fully abolish the formation of lipid droplets (Fig. 6A). The expression of *Cebpb* and *Ppara* genes was significantly reduced in C3H10T1/2-*Egr1* cells compared to control cells as that of *Cidea*, *Plin5*, *Pank1*, *Ogdh and Sucla2* genes (Fig. 6C). This showed that beige differentiation and the heat-producing ability of C3H10T1/2 cells were impaired upon *Egr1* overexpression. *Egr1* overexpression also blocked white adipocyte differentiation in C3H10T1/2 cells (see Supplementary Fig. 7), as previously observed^32^. The inhibition of both beige and white differentiation programs by EGR1 is to be related with the direct (*Cebpb*) and indirect transcriptional regulation of generic adipogenesis genes by EGR1 (Fig. 3B–D).

In order to assess whether EGR1 promotes the expression of ECM genes in mesenchymal stem cells in the context of adipocyte differentiation, we analysed the expression of *Col5a2*, *Fn1* and *Postn* in C3H10T1/2 and C3H10T1/2-*Egr1* cells during beige (Fig. 6D) and white (see Supplementary Fig. 7) adipocyte differentiation. The expression of *Col5a2*, *Fn1* and *Postn* genes was upregulated in *Egr1* overexpressing cells, showing that EGR1 activated the expression of ECM genes during adipocyte differentiation. The positive regulation of ECM genes by EGR1 during adipocyte differentiation was consistent with similar regulation in the context of fibrosis, atherosclerosis and tendon repair^32,52^. We conclude that forced EGR1 expression in mouse mesenchymal stem cells reduces beige marker expression, while promoting ECM gene expression.

**Figure 7:**
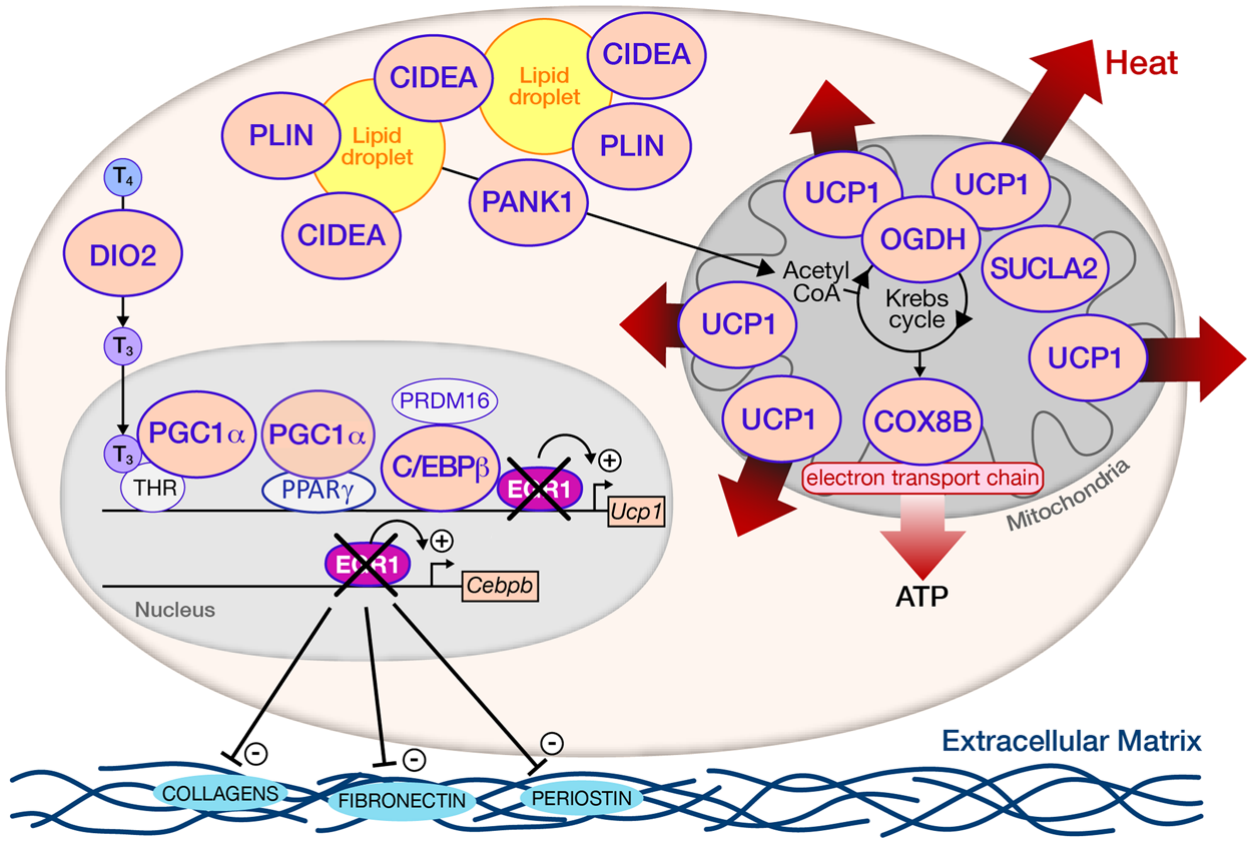
EGR1 regulates beige adipocytes differentiation, metabolism and extracellular matrix formation. *Egr1* loss-of-function upregulates the expression of genes encoding C/EBPß, PGC1α, UCP1, COX8B, SUCLA2, OGDH, CIDEA, PLIN5, PANK1 and DIO2, leading to a significant browning of SC-WAT in *Egr1* ^−/−^ mice. *Egr1* deletion downregulates the expression of genes encoding the ECM proteins Collagens, Fibronectin and Perisotin. Our study confirms the opposite correlation between adipose tissue browning and fibrogenesis.

In summary, the deletion of *Egr1* induces WAT browning by the release of the EGR1-mediated repression of the *Cebpb* and *Ucp1* promoters (Fig. 7A,B). *Egr1* loss-of-function causes the overexpression of the beige adipocyte differentiation genes *Cebpb* and *Ppargc1*, which both activate the expression of the thermogenic marker *Ucp1* through a recruitment to its promoter^18,53^ (Fig. 7B). In addition, the beige adipocytes metabolic genes *Dio2*, *Cidea*, *Plin*, *Pank1*, *Cox8b*, *Ogdh* and *Sucla2a* are also upregulated (Fig. 7B) to induce the browning of SC-WAT in *Egr1* ^−/−^ mice, without any cold stimulation or fasting. The expression of ECM genes is reduced in the context of Egr1 loss-of-function (Fig. 7B). Reciprocally, *Egr1* gain-of-function represses the expression of *Cebpb* and *Ucp1* presumably through the recruitment of EGR1 to their promoters (Fig. 7C). In addition, the beige adipocyte metabolic genes *Dio2*, *Cidea*, *Plin*, *Pank1*, *Cox8b*, *Ogdh* and *Sucla2a* are also downregulated (Fig. 7C), which prevents the differentiation of mesenchymal stem cells into beige adipocytes. The expression of ECM genes is enhanced in the context of *Egr1* gain-of-function (Fig. 7C).

The upregulated expression profile of beige differentiation markers and downregulated profile of ECM genes in *Egr1*-deficient WAT define a molecular signature of beige adipocyte differentiation program and constitute a protective signature against white adipocyte lipid accumulation. This study identifies *Egr1* deficiency as a therapeutic approach to counteract obesity.

## Methods

All experimental procedures using mice were conducted in accordance with the European guidelines (2010/63/UE) and were approved by the French National Ethic Committee for animal experimentation N°05 and are registered under the number 01789.02.

### Mouse lines

The *Egr1* gene was inactivated by homologous recombination with insertion of the *LacZ* coding sequence within the *Egr1* 5′ untranslated region in addition to a frameshift mutation upstream of the DNA-binding domain of *Egr1* ^54^. The line was maintained on a C57BL/6 J background (Janvier, France). All animals were kept under controlled photo-period (lights on 08:00–20:00 hours) and a diet of commercial rodent chow and tap water *ad libitum*.

Age-matched groups of 2-week-old, 1-month-old and 4-month-old *Egr1* ^+/+^ and *Egr1* ^−/−^ female mice derived from heterozygous intercrosses of *Egr1* ^+/−^ were used for the RNA-sequencing, RT-qPCR and immunostaining experiments.

For the RNA-sequencing experiments, we used 3 *Egr1* ^+/+^ and 3 *Egr1* ^−/−^ female mice from 4 different litters. Among these animals, 1 *Egr1* ^+/+^ and 1 *Egr1* ^−/−^ mice were littermates.

For the RT-qPCR experiments, we used 5 *Egr1* ^+/+^ and 5 *Egr1* ^−/−^ 2-week-old female mice from 7 different litters. Among them, 1 *Egr1* ^+/+^ and 1 *Egr1* ^−/−^ mice were littermates. We used 6 *Egr1* ^+/+^ and 5 *Egr1* ^−/−^ 4-month-old female mice from 8 different litters.

For the immunostainings experiments, we used 3 *Egr1* ^+/+^ and 2 *Egr1* ^−/−^ 1-month-old female mice. Among them, 2 *Egr1* ^+/+^ and 2 *Egr1* ^−/−^ were littermates. We used 3 *Egr1* ^+/+^, 3 *Egr1* ^−/−^ and 2 *Egr1* ^+/−^ 4-month-old female mice. Among these mice, 1 *Egr1* ^+/+^, 1 *Egr1* ^−/−^ and 1 *Egr1* ^+/−^ were littermates.

### *In situ* hybridization to adipose tissue sections

Inguinal subcutaneous fat pads were isolated from 1-month-old female mice, fixed in 4% paraformaldehyde overnight and processed for *in situ* hybridization to 6 mm wax tissue sections as previously described^55^. The digoxigenin-labeled mouse *Egr1* probe was used as previously described^54^.

### RNA isolation, sequencing and transcriptomic analysis

Fresh inguinal subcutaneous fat pads were removed from 2-week-old euthanized *Egr1* ^+/+^ (N = 3) and *Egr1* ^−/−^ (N = 3) female mice and homogenized using a mechanical disruption device (Lysing Matrix A, Fast Prep MP1, 4 × 30 s, 6 m.s^−1^). Total RNA was isolated using the RNeasy mini kit (Qiagen) with 15 min of DNase I (Qiagen) treatment according to the manufacturer’s protocol. Preparation of cDNA libraries and sequencing was performed at the “Ecole Normale Supérieure” Genomic Platform (Paris, France). Ribosomal RNA depletion was performed with the Ribo-Zero kit (Epicentre), using 500 ng of total RNA. Libraries were prepared using the strand specific RNA-Seq library preparation ScriptSeq V2 kit (Epicentre). 51-bp paired-end reads were generated using a HiSeq. 1500 device (Illumina). A mean of 56.9 ± 6.3 million reads passing the Illumina quality filter were obtained for each of the 6 samples. Reads were mapped against the *mus musculus* reference genome (UCSC Dec. 2011, GRCm38/mm10) using TopHat v2.1.0^56^, Bowtie (v2.2.5)^57^, and the Release M8 (GRCm38.p4) GTF annotations as a guide. Read counts were assigned to gene features using Feature Counts v1.4.6.p5^58^ and differential expression analysis was performed with DESeq. 2 v1.6.3^59^. Full details of the Galaxy workflow used in this study can be retrieved via the following link: https://mississippi.snv.jussieu.fr/u/emmanuellehavis/w/copy-of-grasostendon-differential-expression-2. Gene Ontology analysis on differentially expressed genes (Padj < 0.05) was performed with DAVID Bioinformatic Resources 6.8^60^. Sequencing data was uploaded to the Gene Expression Omnibus (GEO) database under the accession number GSE91058.

### RNA isolation, Reverse-Transcription and quantitative real time PCR

Fresh inguinal subcutaneous fat pads were removed from 2-week-old and 4-month-old euthanized *Egr1* ^+/+^ and *Egr1* ^−/−^ female mice and homogenized using a mechanical disruption device (Lysing Matrix A, Fast Prep MP1, 4 × 30 s, 6 m.s^−1^). Total RNA was isolated using the RNeasy mini kit (Qiagen) with 15 min of DNase I (Qiagen) treatment according to the manufacturer’s protocol.

For RT-qPCR analyses, 500 ng RNA was Reverse-Transcribed using the High Capacity Retrotranscription kit (Applied Biosystems). Quantitative PCR was performed using SYBR Green PCR Master Mix (Applied Biosystems) using primers listed in Supplementary Table 1. We used *Actb* as housekeeping gene for the analysis of the SC-WAT from 2-week-old mice and *Rplp0* for the analysis of the SC-WAT from 4-month-old mice and C3H10T1/2 and C3H10T1/2-*Egr1* cells. The relative mRNA levels were calculated using the 2^−ΔΔCt^ method^61^. The ΔCt values were obtained by calculating the differences: Ct(gene of interest) – Ct(housekeeping gene) in each sample. We obtained the ΔΔCt values by calculating the differences between ΔCt(Experiment) and the average of ΔCt(control) values. For mRNA level analysis in SC-WAT, *Egr1* ^−/−^ values were considered as experimental and *Egr1* ^+/+^ as controls. The controls were normalized to 1.

5 to 6 independent RNA samples of 2-week-old and 4-month-old *Egr1* ^+/+^ and *Egr1* ^−/−^ female mice were analysed in duplicate. For mRNA level analysis in cell cultures, C3H10T1/2-*Egr1* values were considered as experimental and C3H10T1/2 as controls. 6 independent RNA samples were analysed in duplicate for each time point.

### Chromatin Immunoprecipitation

ChIP assays were performed with previously reported protocol^62^ on the inguinal subcutaneous adipose tissue isolated from 60 2-week-old mice, homogenized using a mechanical disruption device (Lysing Matrix A, Fast Prep MP1, 3 × 30 sec). 8 µg of the rabbit polyclonal anti-Egr-1/Krox24 (C-19) antibody (Santa Cruz Biotechnology) or 8 µg of the goat anti-mouse IgG2b (Southern biotechnology) were used to immunoprecipitate 30 µg of sonicated chromatin. ChIP products were analyzed by quantitative PCR. 15 µg of chromatin was isolated before chromatin immunoprecipitation, to be used as positive control for the PCR experiments (Input). ChIP products and Inputs were analyzed by quantitative PCR to amplify the promoter regions upstream the *Cebpb* (−660 bp; −530 bp), *Ppargc1a* (−860 bp; −730 bp), *Ucp1* (−170 bp; + 20 bp) *and Gapdh* (−2,9 Kb; −2,7 Kb; negative control) coding sequences. qPCR amplicons were loaded on a 1% agarose gel. The primer list is displayed in Supplementary Table 1.

### Immunohistochemistry

Fresh inguinal subcutaneous fat pads were removed from 1-month-old and 4-month-old euthanized *Egr1* ^+/+^ and *Egr1* ^−/−^ female mice, fixed in 4% formaldehyde overnight at 4 °C and processed for immunohistochemistry on 12 µm wax tissue sections, as previously described^63^. After wax removal, for UCP1 immunodetection, heat-induced epitope retrieval was performed by incubating sections 5 min at 95 °C in Glycine-HCl buffer (0.05 M Glycine, pH3.5). UCP1 protein was detected using rabbit polyclonal antibody (1:200, ab10983, Abcam), followed by secondary anti-rabbit HRP conjugate antibody (1:200, 170-6515, Biorad) and DiaminoBenzidine Tetra-Hydrochloride protocol (DAB) staining or followed by secondary anti-rabbit fluorescent antibody (1:200, Goat anti-Rabbit Alexa 488, A11008, Invitrogen) staining.

For fibronectin immunodetection, heat-induced epitope retrieval was performed by incubating sections 7 min at 95 °C in citrate buffer (10 mM, pH 6). Fibronectin protein was detected using mouse monoclonal antibody (1:200, F7387, Sigma), followed by secondary anti-mouse fluorescent antibody (1:200, Goat anti-Mouse Alexa 555, A21422, Invitrogen) staining.

Nuclei were visualised either by Hematoxylin & Eosin (H&E) histological staining using a standard protocol or by DAPI staining according to manufacturer’s instructions (DAPI, D9542, Sigma).

C3H10T1/2 and C3H10T1/2-Egr1 cells were cultured in beige or white adipocyte differentiation medium for 8 and 10 days, respectively, on cover slips. Cells were fixed with 4% Paraformaldehyde (Sigma) for 15 min. UCP1 protein was detected using rabbit polyclonal antibody (1:200, ab10983, Abcam), followed by secondary anti-rabbit HRP conjugate antibody (1:200, 170-6515, Biorad) and DiaminoBenzidine Tetra-Hydrochloride protocol (DAB) staining. Hematoxylin & Eosin (H & E) histological staining was performed using a standard protocol.

### Cell number measurements

All cell number measurements were performed using the free software ImageJ (Rasband, W.S., ImageJ, U. S. National Institutes of Health, Bethesda, Maryland, USA, http://imagej.nih.gov/ij/, 1997–2012).

To quantify the number of white and beige adipocytes in SC-WAT of *Egr1* ^+/++/+^ and *Egr1* ^−/−^ mice, cell number was counted per arbitrary unit area on sections originating from 1-month-old and 4-month-old female mice. The beige adipocytes were identified by the expression of UCP1 detected by immunodetection with DAB staining or fluorescent staining and by the multilocular aspect of the lipid droplets combined with positive Hematoxylin or DAPI nucleus staining. The white adipocytes were identified both by a unilocular lipid droplet and positive DAPI staining.

The number of beige and white cells in 1-month-old mice was counted from 10 sections originating from 6 fat pads of 2 *Egr1* ^+/+^ mice and from 11 sections originating from 4 fat pads of 2 *Egr1* ^−/−^ mice. The number of beige and white cells in 4-month-old mice was counted from 13 sections originating from 6 fat pads of 3 *Egr1* ^+/+^ mice, from 14 sections originating from 6 fat pads of 3 *Egr1* ^−/−^ mice and from 8 sections originating from 4 fat pads of 2 *Egr1* ^+/−^ mice.

To quantify the total number of cells in SC-WAT of *Egr1* ^+/+^ and *Egr1* ^−/−^ mice, the number of DAPI + cells was counted per arbitrary unit area on sections originating from 1-month-old and 4-month-old female mice. The number of DAPI + cells in 1-month-old mice was counted from 9 sections originating from 6 fat pads of 3 *Egr1* ^+/+^ mice and from 11 sections originating from 4 fat pads of 2 *Egr1* ^−/−^ mice. The number of DAPI + cells in 4-month-old mice was counted from 11 sections originating from 6 fat pads of 3 *Egr1* ^+/+^ mice, from 12 sections originating from 6 fat pads of 3 *Egr1* ^−/−^ mice and from 8 sections originating from 4 fat pads of 2 *Egr1* ^+/−^ mice.

To quantify the number of C3H10T1/2 and C3H10T1/2-Egr1 cells at Day 0 and Day 8 of beige adipocyte differentiation conditions the number of Hematoxylin-positive cells was counted per unit area from 10 wells for each condition.

### Cell cultures

Mouse mesenchymal stem cells, C3H10T1/2^64^ and the stable *Egr1* overexpressing counterparts, C3H10T1/2-Egr1^32^ cells, were plated to 6-well plates at a density of 330,000 cells/well and grown in Dulbecco’s Modified Eagle’s Medium (DMEM, Invitrogen) supplemented with 10% foetal bovine serum (FBS, Sigma), 1% penicillin-streptomycin (Sigma), 1% Glutamin (Sigma), 800 μg/ml G418 Geneticin (Sigma) and incubated at 37 °C in humidified atmosphere with 5% CO_2_.

Confluent cells were cultured in beige differentiation induction medium for 2 days and in beige maturation medium for 6 days according to published protocols^65^. Day 0 corresponds to the addition of beige differentiation induction medium on confluent cells. Beige differentiation induction medium includes DMEM, 10% FBS, 1% penicillin-streptomycin, 10 µg/mL Insulin (Sigma), 0.25 µM Dexamethasone (Sigma), 0.5 mM 3-Isobutyl-1-methylxanthine (IBMX, Sigma), 50 nM 3.3′,5-Triiodo-L-thyronine sodium salt (T_3_, Sigma), 20 µM Curcumin (Sigma). The beige maturation medium comprises DMEM, 10% FBS, 1% penicillin-streptomycin, 10 µg/mL Insulin (Sigma), 50 nM 3,3′,5-Triiodo-L-thyronine sodium salt (T_3_, Sigma), 20 µM Curcumin (Sigma), 1 µM Rosiglitazone (Sigma). The maturation medium was changed every 2 days. Cells subjected to beige adipocyte differentiation medium were fixed for histological analysis or lysed for gene expression analysis at Day 0, Day 1, Day 6 and Day 8.

Confluent cells were cultured in white differentiation induction medium for 2 days and in white maturation medium for 8 days. Day 0 corresponds to the addition of white differentiation medium. White differentiation induction medium includes DMEM, 10% FBS, 1% penicillin-streptomycin, 10 µg/mL Insulin (Sigma), 0.25 µM Dexamethasone (Sigma), 0.5 mM 3-Isobutyl-1-methylxanthine (IBMX, Sigma), 30 nM 3.3′,5-Triiodo-L-thyronine sodium salt (T_3_, Sigma). The white maturation medium comprises DMEM, 10% FBS, 1% penicillin-streptomycin and 10 µg/mL Insulin (Sigma). The maturation medium was changed every 2 days. Cells subjected to white adipocyte differentiation medium were stopped at Day 0, Day 1, Day 4 and Day 10 for histological and gene expression analysis by RT-qPCR.

### Oil Red O staining

C3H10T1/2 and C3H10T1/2-Egr1 cells were cultured in beige or white adipocyte differentiation medium for 8 and 10 days, respectively. Cells were fixed with 4% Paraformaldehyde (Sigma) for 15 min and washed twice with excess distilled H_2_O (Millipore). 60% Isopropanol was added for 5 min and replaced with an Oil Red O (Sigma) staining mixture, consisting of Oil Red O solution (0.5% Oil Red O dye in Isopropanol) and water in a 6:4 ratio, for 15 min. Cells were rinsed three times in distilled H_2_O, followed by a standard Hematoxylin & Eosin staining protocol.

### Statistical analyses

Data was analysed using the non-parametric Mann-Withney test or ANOVA test with Graphpad Prism V6. Results are shown as means ± standard deviations. The p-values are indicated either with the value or with * or #.

## Electronic supplementary material

Supplementary figures
