# Supplementary material for: Egr1 deficiency induces browning of inguinal subcutaneous white adipose tissue in mice
Source: Sci Rep. 2017 Nov 23;7:16153. doi: 10.1038/s41598-017-16543-7 (PMC5701004; doi:10.1038/s41598-017-16543-7)
Supplement: Supplementary file 1 — Supplementary figures [file 41598_2017_16543_MOESM1_ESM.pdf]

**Supplementary information**

# Egr1 deficiency induces browning of inguinal subcutaneous white adipose tissue in mice

Cécile Milet[1*$], Marianne Bléher[1$], Kassandra Allbright[2], Mickael Orgeur[1], Fanny Coulpier[3], Delphine Duprez[1#*], Emmanuelle Havis[1#*]

[1] Sorbonne Universités, UPMC Univ Paris 06, CNRS UMR7622, Inserm U1156, IBPS-Developmental Biology Laboratory, F-75005 Paris, France.

[2] University of Pittsburgh, Pittsburgh, Pennsylvania, United States.

[3] École normale supérieure, PSL Research University, CNRS, Inserm, Institut de Biologie de l'École normale supérieure (IBENS), Plateforme Génomique, 75005 Paris, France.

[$] Co-first authors

[#] Co-senior authors

* Corresponding authors:

Emmanuelle Havis, emmanuelle.havis@upmc.fr, +33 1 44 27 34 40

Delphine Duprez, delphine.duprez@upmc.fr, +33 1 44 27 27 53

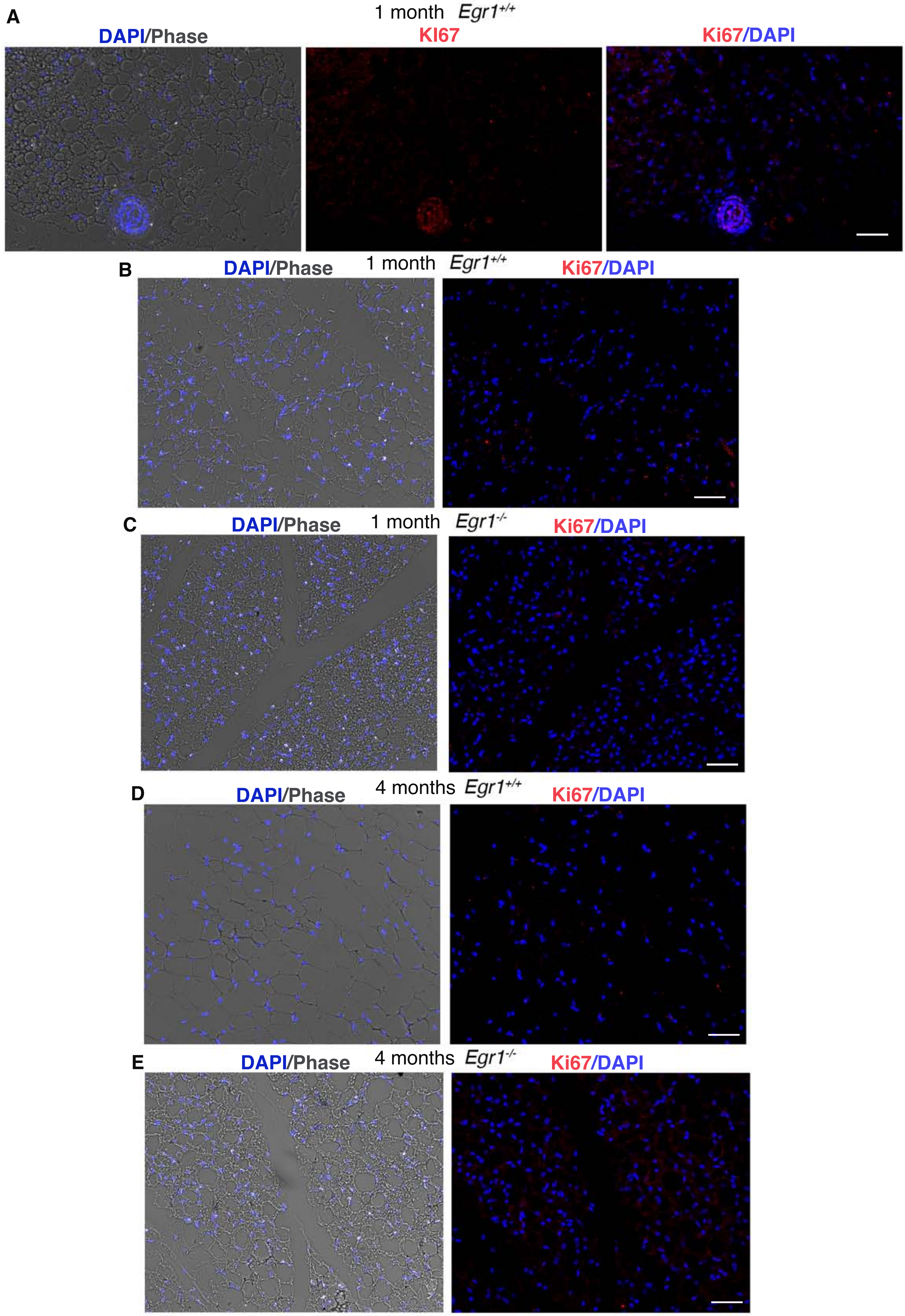
A
1 month Egr1+/+
DAPI/Phase
KI67
Ki67/DAPI
B
DAPI/Phase
1 month Egr1+/+
Ki67/DAPI
C
DAPI/Phase
1 month Egr1-/-
Ki67/DAPI
D
DAPI/Phase
4 months Egr1+/+
Ki67/DAPI
E
DAPI/Phase
4 months Egr1-/-
Ki67/DAPI

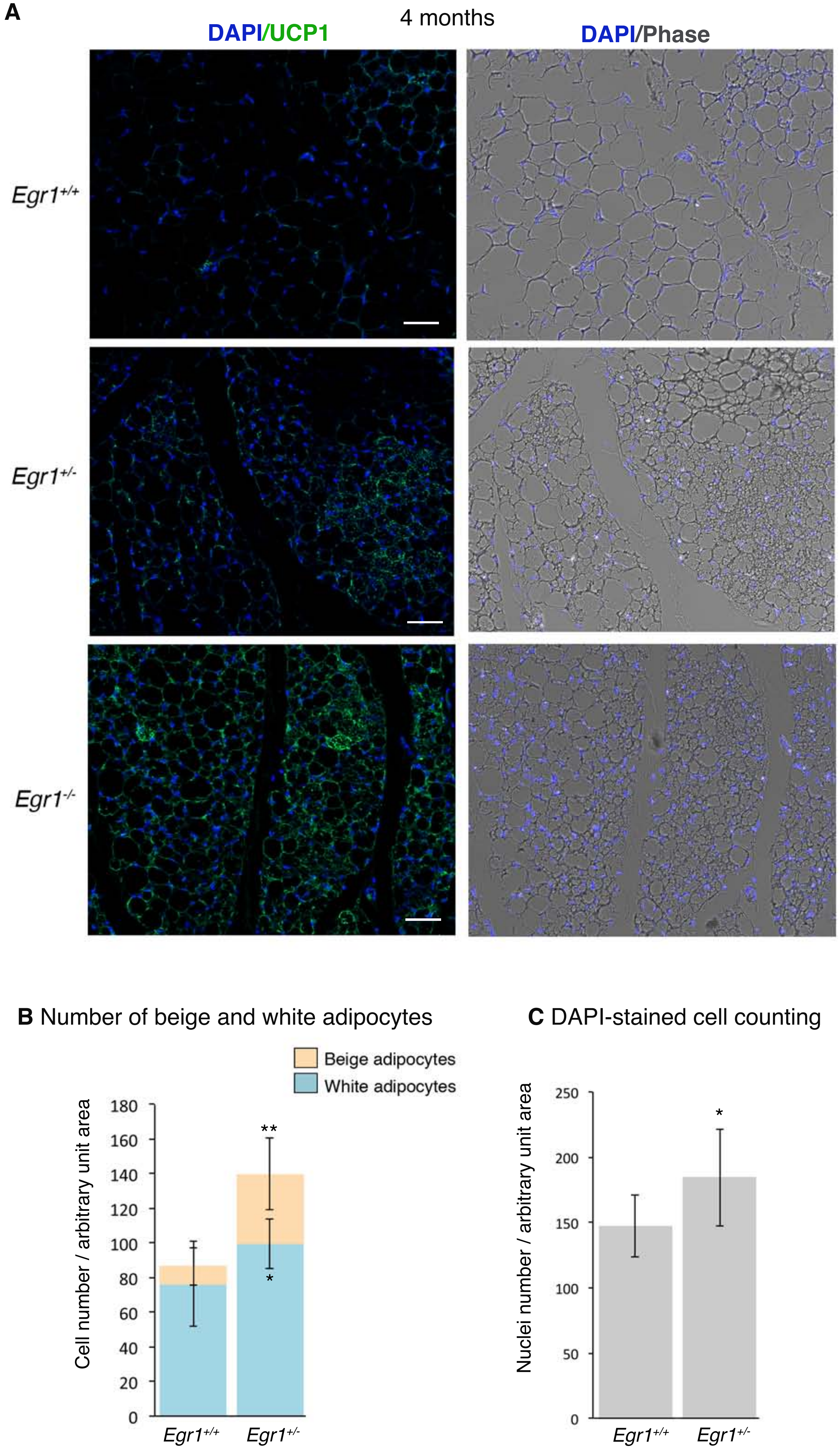
A
4 months
DAPI/UCP1
DAPI/Phase
Egr1+/+
Egr1+/-
Egr1-/-
B Number of beige and white adipocytes
Beige adipocytes
White adipocytes
Cell number / arbitrary unit area
180
160
140
120
100
80
60
40
20
0
**
*
Egr1+/+
Egr1+/-
C DAPI-stained cell counting
Nuclei number / arbitrary unit area
250
200
150
100
50
0
*
Egr1+/+
Egr1+/-

**List of up-regulated genes in the ingWAT of postnatal Egr1-/- mice**

| Contig | log2FoldChar | Fold Change | padj | Description |
|---|---|---|---|---|
| **Genes expressed in adipose tissue derived- mesenchymal stem cells** | | | | |
| *Gnas* | 0.471 | 1.386 | 3.94E-02 | GNAS (guanine nucleotide binding protein, alpha stimulating) complex locus |
| | | | | |
| **Genes expressed during adipocytes (white/beige) differentiation** | | | | |
| *Cebpb* | 0.583 | 1.498 | 2.03E-02 | CCAAT/enhancer binding protein (C/EBP), beta |
| *Ppara* | 0.682 | 1.604 | 9.66E-03 | peroxisome proliferator activated receptor alpha |
| | | | | |
| Genes expressed in beige differenciated adipocytes: metabolism and thermogenic genes | | | | |
| *Acacb* | 0.747 | 1.679 | 2.57E-03 | acetyl-Coenzyme A carboxylase beta |
| *Acadm* | 0.508 | 1.422 | 3.32E-02 | acyl-Coenzyme A dehydrogenase, medium chain |
| *Acadvl* | 0.504 | 1.418 | 3.47E-02 | acyl-Coenzyme A dehydrogenase, very long chain |
| *Aco2* | 0.628 | 1.546 | 6.26E-03 | aconitase 2, mitochondrial |
| *Acot11* | 1.171 | 2.251 | 1.92E-07 | acyl-CoA thioesterase 11 |
| *Apoc1* | 0.605 | 1.521 | 9.48E-03 | apolipoprotein C-I |
| *bscl2* | 0.479 | 1.394 | 2.18E-02 | BSCL2, seipin lipid droplet biogenesis associated |
| *Chchd10* | 0.502 | 1.416 | 4.64E-02 | coiled-coil-helix-coiled-coil-helix domain containing 10 |
| *Cidea* | 0.818 | 1.763 | 6.35E-04 | cell death-inducing DNA fragmentation factor, alpha subunit-like effector A |
| *Cisd3* | 0.664 | 1.584 | 1.91E-02 | CDGSH iron sulfur domain 3 |
| *Clstn3* | 0.725 | 1.653 | 1.78E-02 | calsyntenin 3 |
| *Cluh* | 0.552 | 1.466 | 1.01E-02 | clustered mitochondria (cluA/CLU1) homolog |
| *Coq6* | 0.684 | 1.607 | 4.08E-03 | coenzyme Q6 monooxygenase |
| *Cox5a* | 0.694 | 1.618 | 2.10E-03 | cytochrome c oxidase subunit Va |
| *Cox8b* | 0.670 | 1.591 | 2.10E-03 | cytochrome c oxidase subunit VIIIb |
| *Cpn2* | 0.809 | 1.752 | 5.18E-03 | carboxypeptidase N, polypeptide 2 |
| *Cpt1b* | 0.620 | 1.537 | 2.54E-02 | carnitine palmitoyltransferase 1b, muscle |
| *Cs* | 0.645 | 1.563 | 3.97E-03 | citrate synthase] [Gene Type: protein-coding] |
| *Cspg4* | 0.563 | 1.478 | 2.63E-02 | chondroitin sulfate proteoglycan 4 |
| *Cyc1* | 0.486 | 1.401 | 4.44E-02 | cytochrome c-1 |
| *Dhrs11* | 0.804 | 1.746 | 2.96E-03 | dehydrogenase/reductase (SDR family) member 11 |

| | | | | |
|---|---|---|---|---|
| *Dhrs9* | 0.971 | 1.961 | 9.52E-04 | dehydrogenase/reductase (SDR family) member 9 |
| *Dio2* | 1.421 | 2.677 | 4.63E-09 | deiodinase, iodothyronine, type II |
| *Elovl6* | 0.536 | 1.450 | 4.30E-02 | ELOVL family member 6, elongation of long chain fatty acids (yeast)] |
| *Etfa* | 0.525 | 1.439 | 2.03E-02 | electron transferring flavoprotein, alpha polypeptide |
| *Etfdh* | 0.506 | 1.420 | 3.34E-02 | electron transferring flavoprotein, dehydrogenase |
| *Gpd1* | 0.675 | 1.596 | 5.58E-03 | glycerol-3-phosphate dehydrogenase 1 (soluble) |
| *Gpd2* | 0.783 | 1.721 | 5.70E-04 | glycerol phosphate dehydrogenase 2, mitochondrial |
| *Hadhb* | 0.642 | 1.561 | 5.77E-03 | hydroxyacyl-Coenzyme A dehydrogenase, beta subunit |
| *Idh3a* | 0.586 | 1.501 | 1.02E-02 | isocitrate dehydrogenase 3 (NAD+) alpha |
| *Idh3g* | 0.497 | 1.411 | 2.36E-02 | isocitrate dehydrogenase 3 (NAD+), gamma |
| *Kcnk3* | 0.625 | 1.543 | 1.13E-02 | potassium channel, subfamily K, member 3 |
| *Kng2* | 0.785 | 1.723 | 8.74E-03 | kininogen 2 |
| *Ldhb* | 0.719 | 1.646 | 2.83E-03 | lactate dehydrogenase B |
| *Letmd1* | 0.747 | 1.679 | 1.41E-03 | LETM1 domain containing 1 |
| *Lrg1* | 0.659 | 1.579 | 1.50E-02 | leucine-rich alpha-2-glycoprotein 1 |
| *Mdh2* | 0.565 | 1.479 | 1.62E-02 | malate dehydrogenase 2, NAD (mitochondrial) |
| *Mecr* | 0.547 | 1.461 | 1.04E-02 | mitochondrial trans-2-enoyl-CoA reductase |
| *Mlxipl* | 0.706 | 1.632 | 3.11E-03 | MLX interacting protein-like |
| *Mrap* | 0.684 | 1.606 | 1.42E-03 | melanocortin 2 receptor accessory protein |
| *Nampt* | 0.678 | 1.600 | 1.42E-03 | nicotinamide phosphoribosyltransferase |
| *Nrg4* | 0.454 | 1.370 | 2.75E-02 | neuregulin 4 |
| *Ntrk3* | 0.619 | 1.536 | 8.33E-03 | neurotrophic tyrosine kinase, receptor, type 3 |
| *Nudt7* | 0.494 | 1.409 | 4.56E-02 | nudix (nucleoside diphosphate linked moiety X)-type motif 7 |
| *Ogdh* | 0.653 | 1.572 | 2.81E-03 | oxoglutarate (alpha-ketoglutarate) dehydrogenase (lipoamide) |
| *Oplah* | 0.563 | 1.477 | 7.75E-03 | 5-oxoprolinase (ATP-hydrolysing) |
| *Otop1* | 0.748 | 1.679 | 8.21E-04 | otopetrin 1 |
| *Pald1* | 0.491 | 1.405 | 2.54E-02 | phosphatase domain containing, paladin 1 |
| *Pank1* | 0.815 | 1.759 | 1.97E-04 | pantothenate kinase 1 |
| *Plin5* | 0.817 | 1.762 | 1.46E-04 | perilipin 5 |
| *Pm20d1* | 1.052 | 2.074 | 2.36E-04 | peptidase M20 domain containing 1 |
| *Ppargc1a* | 0.870 | 1.827 | 6.30E-04 | peroxisome proliferative activated receptor, gamma, coactivator 1 alpha |
| *Ppargc1b* | 0.792 | 1.732 | 3.45E-04 | peroxisome proliferative activated receptor, gamma, coactivator 1 beta |

| | | | | |
|---|---|---|---|---|
| *Ptp4a1* | 0.445 | 1.362 | 3.55E-02 | protein tyrosine phosphatase 4a1 |
| *Sh2b2* | 0.621 | 1.538 | 5.44E-03 | SH2B adaptor protein 2 |
| *Slc25a20* | 0.540 | 1.454 | 3.06E-02 | solute carrier family 25 (mitochondrial carnitine/acylcarnitine translocase), member 20 |
| *Slc25a22* | 0.611 | 1.527 | 2.35E-02 | solute carrier family 25 (mitochondrial carrier, glutamate), member 22 |
| *Slc25a42* | 0.860 | 1.815 | 1.26E-06 | solute carrier family 25, member 42 |
| *Slc25a51* | 0.539 | 1.453 | 8.47E-03 | solute carrier family 25, member 51 |
| *Slc36a2* | 0.750 | 1.682 | 1.70E-04 | solute carrier family 36 (proton/amino acid symporter), member 2 |
| *Slc4a4* | 0.490 | 1.404 | 6.12E-03 | solute carrier family 4 (anion exchanger), member 4 |
| *Sucla2* | 0.626 | 1.543 | 8.09E-03 | succinate-Coenzyme A ligase, ADP-forming, beta subunit |
| *Suclg1* | 0.541 | 1.455 | 2.33E-02 | succinate-CoA ligase, GDP-forming, alpha subunit] |
| *Ucp1* | 1.050 | 2.071 | 1.18E-07 | uncoupling protein 1 (mitochondrial, proton carrier) |

## Genes expressed in White adipose metabolism

| | | | | |
|---|---|---|---|---|
| *Adra1a* | 1.056 | 2.079 | 2.95E-08 | adrenergic receptor, alpha 1a |
| *Adrbk2* | 0.641 | 1.559 | 4.79E-03 | adrenergic, beta, receptor kinase 2 |
| *Chst3* | 0.588 | 1.503 | 4.61E-02 | carbohydrate (chondroitin 6/keratan) sulfotransferase 3 |
| *Cmtm4* | 0.550 | 1.464 | 1.84E-02 | CKLF-like MARVEL transmembrane domain containing 4 |
| *Cntfr* | 0.723 | 1.651 | 2.22E-02 | ciliary neurotrophic factor receptor |
| *Cntnap1* | 0.656 | 1.576 | 4.44E-02 | contactin associated protein-like 1 |
| *Crat* | 0.504 | 1.418 | 1.90E-02 | carnitine acetyltransferase |
| *Ctse* | 1.659 | 3.157 | 2.07E-10 | cathepsin E |
| *Dcun1d3* | 0.512 | 1.426 | 4.08E-02 | DCN1, defective in cullin neddylation 1, domain containing 3 |
| *Dgat2* | 0.647 | 1.566 | 1.19E-02 | diacylglycerol O-acyltransferase 2 |
| *Gk* | 1.074 | 2.106 | 1.74E-09 | glycerol kinase |
| *Gys1* | 0.553 | 1.468 | 1.97E-02 | glycogen synthase 1, muscle |
| *Helz2* | 0.518 | 1.432 | 1.97E-02 | helicase with zinc finger 2, transcriptional coactivator |
| *Hk2* | 0.804 | 1.746 | 3.42E-04 | hexokinase 2 |
| *Mif4gd* | 0.509 | 1.423 | 3.01E-02 | MIF4G domain containing/serum leptin-interacting protein 1 |
| *Pdp2* | 0.583 | 1.498 | 2.75E-02 | pyruvate dehyrogenase phosphatase catalytic subunit 2 |
| *Pnpla3* | 0.819 | 1.764 | 6.72E-04 | patatin-like phospholipase domain containing 3 |
| *Ppip5k1* | 0.531 | 1.445 | 2.06E-02 | diphosphoinositol pentakisphosphate kinase 1 |
| *Ppp1r10* | 0.462 | 1.377 | 4.01E-02 | protein phosphatase 1, regulatory subunit 10 |

| | | | | |
|---|---|---|---|---|
| *Rtn4ip1* | 0.531 | 1.445 | 4.47E-02 | reticulon 4 interacting protein 1 |
| *Syt12* | 0.777 | 1.714 | 1.13E-02 | synaptotagmin XII |
| *Tfrc* | 1.189 | 2.280 | 1.18E-07 | transferrin receptor |
| *Tysnd1* | 0.553 | 1.467 | 4.20E-02 | trypsin domain containing 1 |
| **Genes involved in inflammation in adipose tissue** | | | | |
| *Cdkn1a* | 0.654 | 1.574 | 3.55E-02 | cyclin-dependent kinase inhibitor 1A (P21) |
| *Ifit1* | 0.795 | 1.735 | 2.54E-02 | interferon-induced protein with tetratricopeptide repeats 1 |
| *Il15ra* | 0.483 | 1.398 | 3.95E-02 | interleukin 15 receptor, alpha chain |
| *Il2rb* | 0.810 | 1.753 | 1.43E-02 | interleukin 2 receptor, beta chain |
| *Irf7* | 0.702 | 1.626 | 2.54E-02 | interferon regulatory factor 7 |
| *Tmem38b* | 0.501 | 1.415 | 4.29E-02 | transmembrane protein 38B |
| *Tob1* | 0.524 | 1.438 | 5.25E-03 | transducer of ErbB-2.1 |
| *Tob2* | 0.698 | 1.623 | 2.79E-03 | transducer of ERBB2, 2 |
| *Traf4* | 0.591 | 1.506 | 4.93E-02 | TNF receptor associated factor 4 |
| *Usp2* | 0.642 | 1.560 | 2.59E-02 | ubiquitin specific peptidase 2 |
| **Genes expressed in other cell types within the adipose tissue** | | | | |
| *Adam11* | 0.633 | 1.550 | 2.79E-02 | a disintegrin and metallopeptidase domain 11 |
| *Bst2* | 0.705 | 1.630 | 3.55E-02 | bone marrow stromal cell antigen 2 |
| *Cnnm2* | 0.614 | 1.530 | 4.39E-02 | cyclin M2 |
| *Cd79a* | 0.672 | 1.594 | 4.61E-02 | CD79A antigen |
| *Cdh2* | 0.611 | 1.527 | 1.73E-02 | cadherin 2 |
| *Csrnp1* | 0.715 | 1.641 | 2.05E-02 | cysteine-serine-rich nuclear protein 1 |
| *Dlat* | 0.505 | 1.419 | 3.96E-02 | dihydrolipoamide S-acetyltransferase (E2 component of pyruvate dehydrogenase complex) |
| *Dlst* | 0.510 | 1.424 | 2.62E-02 | dihydrolipoamide S-succinyltransferase (E2 component of 2-oxo-glutarate complex) |
| *Fggy* | 0.717 | 1.644 | 1.35E-02 | FGGY carbohydrate kinase domain containing |
| *Gramd1b* | 0.566 | 1.480 | 1.40E-02 | GRAM domain containing 1B |
| *Pax5* | 0.687 | 1.610 | 3.11E-02 | paired box 5 |
| *Perm1* | 0.712 | 1.638 | 1.05E-02 | PPARGC1 and ESRR induced regulator, muscle 1 |
| *Sbk1* | 0.594 | 1.509 | 1.13E-02 | SH3-binding kinase 1 |
| *Sec61a2* | 0.536 | 1.450 | 1.90E-02 | Sec61, alpha subunit 2 (S. cerevisiae) |

| | | | | |
|---|---|---|---|---|
| *Trim67* | 1.374 | 2.591 | 2.58E-11 | tripartite motif-containing 67 |

## Genes with unidentified function or Non coding RNA

| | | | | |
|---|---|---|---|---|
| *1110001J03Rik* | 0.744 | 1.675 | 5.77E-03 | |
| *Ctcflos* | 0.673 | 1.594 | 3.80E-03 | CCCTC-binding factor (zinc finger protein)-like, opposite strand (ncRNA) |
| *Fam126b* | 0.440 | 1.357 | 2.16E-02 | family with sequence similarity 126, member B |
| *Fam210a* | 0.507 | 1.422 | 2.96E-02 | family with sequence similarity 210, member A |
| *Fam73b* | 0.689 | 1.613 | 1.32E-03 | family with sequence similarity 210, member A |
| *Gdap10* | 0.950 | 1.932 | 8.41E-07 | ganglioside-induced differentiation-associated-protein 10 (ncRNA) |
| *Gm10032* | 0.754 | 1.686 | 3.59E-02 | predicted gene 10032 (ncRNA) |
| *Gm37674* | 0.680 | 1.603 | 3.98E-02 | - |
| *Gm37783* | 0.725 | 1.653 | 2.14E-02 | - |
| *Gm38357* | 0.680 | 1.602 | 1.22E-02 | - |
| *Gm42428* | 0.765 | 1.699 | 1.23E-02 | - |
| *Gm42614* | 0.944 | 1.924 | 1.42E-03 | - |
| *Gm42895* | 0.741 | 1.671 | 2.52E-02 | - |
| *Gm43605* | 0.772 | 1.707 | 2.14E-02 | - |
| *Gm8822* | 2.452 | 5.473 | 5.82E-23 | ARP3 actin-related protein 3 homolog pseudogene |
| *Gm9899* | 0.668 | 1.589 | 1.98E-02 | predicted gene 9899 (ncRNA) |
| *RP23-82I13.6* | 0.813 | 1.757 | 1.34E-03 | - |

| GOTERM_BP_DIRECT | **Up regulated genes in the inguinal subcutaneous adipose tissue of 2-week-old *Egr1*$^{-/-}$ mice** | |
|---|---|---|
| | **Enrichment scores** / P-values | **Genes** |
| **NADH Metabolic process** | **95.2/** $3.3^{E}$-11 | *Dlst* dihydrolipoamide S-succinyltransferase<br>*Gpd2* glycerol phosphate dehydrogenase 2, mitochondrial<br>*Gpd1* glycerol-3-phosphate dehydrogenase 1 (soluble)<br>*Idh3a* isocitrate dehydrogenase 3 (NAD+) alpha<br>*Idh3g* isocitrate dehydrogenase 3 (NAD+), gamma<br>*Mdh2* malate dehydrogenase 2, NAD (mitochondrial)<br>*Ogdh* oxoglutarate (alpha-ketoglutarate) dehydrogenase |
| **Tricarboxylic acid cycle** | **53.4/** $1.1^{E}$-13 | *Aco2* aconitase 2, mitochondrial<br>*Cs* citrate synthase<br>*Dlat* dihydrolipoamide S-acetyltransferase<br>*Dlst* dihydrolipoamide S-succinyltransferase<br>*Idh3a* isocitrate dehydrogenase 3 (NAD+) alpha<br>*Idh3g* isocitrate dehydrogenase 3 (NAD+), gamma<br>*Mdh2* malate dehydrogenase 2, NAD (mitochondrial)<br>*Ogdh* oxoglutarate (alpha-ketoglutarate) dehydrogenase<br>*Suclg1* succinate-CoA ligase, GDP-forming, alpha subunit<br>*Sucla2* succinate-Coenzyme A ligase, ADP-forming, beta subunit |
| **Brown fat cell differentiation** | **23.4** /$5.7^{E}$-5 | *Cebpb* CCAAT/enhancer binding protein (C/EBP), beta<br>*Sh2b2* SH2B adaptor protein 2<br>*Lrg1* leucine-rich alpha-2-glycoprotein 1<br>*Mrap* melanocortin 2 receptor accessory protein<br>*Nudt7* nudix -type motif 7<br>*Ucp1* uncoupling protein 1 |
| **Fatty acid metabolic process** | **9.9** /$7.1^{E}$-7 | *Elovl6* elongation of long chain fatty acids, member 6<br>*Acacb* acetyl-Coenzyme A carboxylase beta<br>*Acot11* acyl-CoA thioesterase 11 |

| | | |
|---|---|---|
| | | *Acadm* acyl-Coenzyme A dehydrogenase, medium chain<br>*Acadvl* acyl-Coenzyme A dehydrogenase, very long chain<br>*Crat* carnitine acetyltransferase<br>*Cpt1b* carnitine palmitoyltransferase 1b<br>*Hadhb* hydroxyacyl-Coenzyme A dehydrogenase<br>*Mecr* mitochondrial trans-2-enoyl-CoA reductase<br>*Ppara* peroxisome proliferator activated receptor alpha |
| **Metabolic process** | **4.9**/ 6 .9$^{E}$-7 | *Acacb* acetyl-Coenzyme A carboxylase beta<br>*Aco2* aconitase 2, mitochondrial<br>*Acadm* acyl-Coenzyme A dehydrogenase, medium chain<br>*Acadvl* acyl-Coenzyme A dehydrogenase, very long chain<br>*Dhrs11* dehydrogenase/reductase (SDR family) member 11<br>*Dhrs9* dehydrogenase/reductase (SDR family) member 9<br>*Dlat* dihydrolipoamide S-acetyltransferase<br>*Dlst* dihydrolipoamide S-succinyltransferase<br>*Gys1* glycogen synthase 1, muscle<br>*Hk2* hexokinase 2<br>Hadhb hydroxyacyl-Coenzyme A dehydrogenase<br>Ogdh oxoglutarate (alpha-ketoglutarate) dehydrogenase<br>Pnpla3 patatin-like phospholipase domain containing 3<br>Pm20d1 peptidase M20 domain containing 1<br>Suclg1 succinate-CoA ligase, GDP-forming, alpha subunit)<br>Sucla2 succinate-Coenzyme A ligase, ADP-forming, beta subunit |
| **Lipid metabolic process** | **4.7** /8.3$^{E}$-6 | Bscl2 Berardinelli-Seip congenital lipodystrophy 2<br>Elovl6 elongation of long chain fatty acids, member 6<br>Acacb acetyl-Coenzyme A carboxylase beta<br>Acadm acyl-Coenzyme A dehydrogenase, medium chain<br>Acadvl acyl-Coenzyme A dehydrogenase, very long chain)<br>Crat carnitine acetyltransferase<br>Cpt1b carnitine palmitoyltransferase 1b<br>Cidea cell death-inducing DNA fragmentation factor, alpha subunit-like effector A |

| | | |
|---|---|---|
| | | Dgat2 diacylglycerol O-acyltransferase 2<br>Hadhb hydroxyacyl-Coenzyme A dehydrogenase<br>Mecr mitochondrial trans-2-enoyl-CoA reductase<br>Pnpla3 patatin-like phospholipase domain containing 3<br>Plin5 perilipin 5<br>Ppara peroxisome proliferator activated receptor alpha |

**List of down-regulated genes in the ingWAT of postnatal Egr1-/- mice**

| *Gene symbol* | Log2 (Fold Ch | Fold Change | padj | Description |
|---|---|---|---|---|
| **Extracellular matrix, cytoskeleton and adhesion genes** | | | | |
| *Adamts2* | -0.488 | 0.713 | 3.83E-02 | a disintegrin-like and metallopeptidase (reprolysin type) with thrombospondin type 1 motif, 2 |
| *Adamtsl4* | -0.557 | 0.680 | 4.61E-02 | ADAMTS-like 4 |
| *Adgrd1* | -0.617 | 0.652 | 3.91E-02 | adhesion G protein-coupled receptor D1 |
| *Adgre1* | -0.622 | 0.650 | 1.23E-02 | adhesion G protein-coupled receptor E1 |
| *Antxr1* | -0.520 | 0.697 | 1.51E-02 | anthrax toxin receptor 1 |
| *Aspn* | -0.698 | 0.616 | 1.64E-03 | asporin |
| *Apcdd1* | -0.517 | 0.699 | 4.18E-02 | adenomatosis polyposis coli down-regulated 1 |
| *Cdh11* | -0.555 | 0.681 | 3.46E-02 | cadherin 11 |
| *Ckap4* | -0.552 | 0.682 | 2.14E-02 | cytoskeleton-associated protein 4 |
| *Col14a1* | -0.692 | 0.619 | 2.01E-05 | collagen, type XIV, alpha 1 |
| *Col1a1* | -0.549 | 0.684 | 8.29E-03 | collagen, type I, alpha 1 |
| *Col1a2* | -0.522 | 0.696 | 8.78E-03 | collagen, type I, alpha 2 |
| *Col3a1* | -0.549 | 0.684 | 6.47E-03 | collagen, type III, alpha 1 |
| *Col5a2* | -0.598 | 0.661 | 1.95E-03 | collagen, type V, alpha 2 |
| *Dab2* | -0.555 | 0.681 | 1.76E-02 | disabled 2, mitogen-responsive phosphoprotein |
| *Dbn1* | -0.598 | 0.661 | 4.64E-02 | drebrin 1 |
| *Dcn* | -0.790 | 0.578 | 7.84E-06 | decorin |
| *Ddr2* | -0.548 | 0.684 | 7.24E-03 | discoidin domain receptor family, member 2 |
| *Dpt* | -0.641 | 0.641 | 4.05E-03 | dermatopontin |
| *Dpysl3* | -0.669 | 0.629 | 1.08E-03 | dihydropyrimidinase-like 3 |
| *Ecm2* | -0.541 | 0.688 | 3.83E-02 | extracellular matrix protein 2, female organ and adipocyte specific |
| *Efemp1* | -0.735 | 0.601 | 1.15E-03 | epidermal growth factor-containing fibulin-like extracellular matrix protein 1 |
| *Fap* | -0.600 | 0.660 | 4.05E-03 | fibroblast activation protein |
| *Fbn1* | -0.781 | 0.582 | 7.58E-05 | fibrillin 1 |
| *Fmr1* | -0.504 | 0.705 | 2.61E-02 | fragile X mental retardation syndrome 1 |
| *Fn1* | -0.676 | 0.626 | 2.04E-04 | fibronectin 1 |
| *Fndc1* | -0.535 | 0.690 | 4.21E-02 | fibronectin type III domain containing 1 |
| *Itgbl1* | -0.604 | 0.658 | 4.20E-02 | integrin, beta-like 1 |

| | | | | |
|---|---|---|---|---|
| *Lum* | -0.741 | 0.598 | 1.50E-05 | lumican |
| *Mmp14* | -0.478 | 0.718 | 3.95E-02 | matrix metallopeptidase 14 (membrane-inserted) |
| *Mmp2* | -0.464 | 0.725 | 3.66E-02 | matrix metallopeptidase 2] |
| *Mmp23* | -0.630 | 0.646 | 8.69E-03 | matrix metallopeptidase 23 |
| *Pcdh20* | -0.697 | 0.617 | 2.37E-02 | protocadherin 20 |
| *Pcdhb14* | -0.749 | 0.595 | 5.51E-03 | protocadherin beta 14 |
| *Pcolce2* | -0.771 | 0.586 | 4.73E-03 | procollagen C-endopeptidase enhancer 2 |
| *Postn* | -0.615 | 0.653 | 1.21E-03 | periostin, osteoblast specific factor |
| *Srpx2* | -0.799 | 0.575 | 1.77E-03 | sushi-repeat-containing protein, X-linked 2 |
| *Thbs3* | -0.575 | 0.671 | 2.05E-02 | thrombospondin 3 |
| *Timp2* | -0.537 | 0.689 | 2.54E-02 | tissue inhibitor of metalloproteinase 2 |
| *Tppp3* | -0.667 | 0.630 | 1.26E-02 | tubulin polymerization-promoting protein family member 3 |
| *Vcan* | -0.646 | 0.639 | 1.13E-02 | versican |
| *Vnn3* | -0.663 | 0.632 | 1.94E-02 | vanin 3 |
| **Genes involved in adipose tissue metabolism and adipocyte metabolism (cell signalling/cell cycle/transcription)** | | | | |
| *Aebp1* | -0.710 | 0.611 | 2.34E-04 | Adipocyte Enhancer binding protein 1 |
| *Akr1c14* | -0.858 | 0.552 | 8.06E-04 | aldo-keto reductase family 1, member C14 |
| *Akr1c18* | -0.921 | 0.528 | 1.21E-04 | aldo-keto reductase family 1, member C18 |
| *Alb* | -0.734 | 0.601 | 3.66E-03 | albumin |
| *Aldh1a3* | -0.752 | 0.594 | 2.10E-03 | aldehyde dehydrogenase family 1, subfamily A3 |
| *Atp2b4* | -0.494 | 0.710 | 2.54E-02 | ATPase, Ca++ transporting, plasma membrane 4 |
| *Atpif1* | -0.513 | 0.701 | 1.98E-02 | ATPase inhibitory factor 1 |
| *Casp1* | -0.667 | 0.630 | 3.32E-02 | caspase 1 |
| *Ces1f* | -0.635 | 0.644 | 2.05E-03 | Carboxylesterase 1F |
| *Clec3b* | -0.711 | 0.611 | 8.48E-03 | C-type lectin domain family 3, member b |
| *Cyb5r3* | -0.659 | 0.633 | 3.79E-03 | cytochrome b5 reductase 3 |
| *Cygb* | -0.532 | 0.692 | 2.01E-02 | cytoglobin |
| *Dnm1* | -0.633 | 0.645 | 8.04E-03 | dynamin 1 |
| *Gas7* | -0.735 | 0.601 | 1.91E-04 | growth arrest specific 7 |
| *Gatm* | -0.537 | 0.689 | 3.59E-02 | glycine amidinotransferase (L-arginine:glycine amidinotransferase) |
| *Ggh* | -0.605 | 0.658 | 4.70E-03 | gamma-glutamyl hydrolase |

| | | | | |
|---|---|---|---|---|
| *Ghr* | -0.596 | 0.662 | 3.79E-03 | growth hormone receptor |
| *Glb1l2* | -0.507 | 0.704 | 4.59E-03 | galactosidase, beta 1-like 2 |
| *Gnai1* | -0.472 | 0.721 | 3.65E-02 | guanine nucleotide binding protein (G protein), alpha inhibiting 1 |
| *Gpc3* | -0.487 | 0.714 | 3.25E-02 | glypican 3 |
| *Gulp1* | -0.528 | 0.693 | 3.52E-02 | GULP, engulfment adaptor PTB domain containing 1] |
| *Hmgn3* | -0.683 | 0.623 | 1.97E-02 | high mobility group nucleosomal binding domain 3 |
| *Kdelr3* | -0.670 | 0.629 | 1.56E-02 | KDEL (Lys-Asp-Glu-Leu) endoplasmic reticulum protein retention receptor 3 |
| *Klf14* | -1.097 | 0.468 | 2.34E-04 | Kruppel-like factor 14 |
| *Klhl13* | -0.755 | 0.592 | 2.40E-04 | kelch-like 13 |
| *Lpar1* | -0.578 | 0.670 | 4.70E-03 | lysophosphatidic acid receptor 1 |
| *Lrrc17* | -0.727 | 0.604 | 4.94E-04 | leucine rich repeat containing 17 |
| *Lrrn4cl* | -0.752 | 0.594 | 1.56E-03 | LRRN4 C-terminal like |
| *Mcm6* | -0.593 | 0.663 | 1.50E-02 | minichromosome maintenance complex component 6 |
| *Mfap2* | -0.841 | 0.558 | 3.58E-03 | microfibrillar-associated protein 2 |
| *Mfap4* | -0.832 | 0.562 | 1.69E-03 | microfibrillar-associated protein 4 |
| *Morf4l2* | -0.480 | 0.717 | 2.85E-02 | mortality factor 4 like 2 |
| *Mpz* | -0.558 | 0.679 | 3.91E-02 | myelin protein zero |
| *Mrc1* | -0.615 | 0.653 | 3.79E-03 | mannose receptor, C type 1 |
| *Mrc2* | -0.484 | 0.715 | 3.23E-02 | mannose receptor, C type 2 |
| *Nr5a2* | -0.721 | 0.607 | 2.01E-02 | nuclear receptor subfamily 5, group A, member 2 |
| *Nrk* | -0.733 | 0.601 | 3.11E-03 | Nik related kinase |
| *Nxnl1* | -0.757 | 0.592 | 2.05E-02 | nucleoredoxin-like 1 |
| *Olfml1* | -0.746 | 0.596 | 2.38E-03 | olfactomedin-like 1 |
| *Olfml2b* | -0.654 | 0.636 | 1.57E-02 | olfactomedin-like 2B |
| *Opcml* | -0.716 | 0.609 | 5.75E-03 | opioid binding protein/cell adhesion molecule-like |
| *P4hb* | -0.433 | 0.741 | 2.22E-02 | prolyl 4-hydroxylase, beta polypeptide |
| *Palm* | -0.509 | 0.703 | 4.28E-02 | paralemmin |
| *Peg10* | -0.696 | 0.617 | 2.35E-02 | paternally expressed 10 |
| *Pi15* | -0.614 | 0.653 | 5.75E-03 | peptidase inhibitor 15 |
| *Pi16* | -0.632 | 0.645 | 2.98E-02 | peptidase inhibitor 16 |
| *Plekha4* | -0.746 | 0.596 | 8.18E-04 | pleckstrin homology domain containing, family A (phosphoinositide binding specific) member 4 |
| *Plscr4* | -0.743 | 0.598 | 5.77E-03 | phospholipid scramblase 4 |

| | | | | |
|---|---|---|---|---|
| *Plxdc2* | -0.624 | 0.649 | 5.96E-04 | plexin domain containing 2 |
| *Ptgfrn* | -0.761 | 0.590 | 1.12E-04 | prostaglandin F2 receptor negative regulator |
| *Qpct* | -0.739 | 0.599 | 2.36E-03 | glutaminyl-peptide cyclotransferase (glutaminyl cyclase) |
| *Rab3il1* | -0.556 | 0.680 | 4.84E-02 | RAB3A interacting protein (rabin3)-like 1 |
| *Rab7b* | -0.763 | 0.589 | 2.10E-03 | RAB7B, member RAS oncogene family |
| *Rassf8* | -0.441 | 0.737 | 3.24E-02 | Ras association (RalGDS/AF-6) domain family (N-terminal) member 8 |
| *Rcan2* | -0.716 | 0.609 | 7.24E-03 | regulator of calcineurin 2 |
| *Rcn1* | -0.567 | 0.675 | 2.99E-03 | reticulocalbin 1 |
| *Rcn3* | -0.582 | 0.668 | 3.00E-02 | reticulocalbin 3, EF-hand calcium binding domain |
| *Rnase4* | -0.589 | 0.665 | 2.81E-02 | ribonuclease, RNase A family 4 |
| *S100a10* | -0.487 | 0.714 | 3.46E-02 | S100 calcium binding protein A10 (calpactin) |
| *S100a6* | -0.584 | 0.667 | 2.54E-02 | S100 calcium binding protein A6 (calcyclin) |
| *Scara5* | -0.585 | 0.667 | 1.97E-02 | scavenger receptor class A, member 5 |
| *Sh3d19* | -0.509 | 0.703 | 2.47E-02 | SH3 domain protein D19 |
| *Slc5a3* | -0.692 | 0.619 | 5.61E-03 | solute carrier family 5 (inositol transporters), member 3 |
| *Smarca1* | -0.685 | 0.622 | 1.61E-02 | SWI/SNF related, matrix associated, actin dependent regulator of chromatin, subfamily a, m1 |
| *Sncg* | -0.889 | 0.540 | 1.12E-04 | synuclein, gamma |
| *Srpx2* | -0.799 | 0.575 | 1.77E-03 | sushi-repeat-containing protein, X-linked 2 |
| *Tceal8* | -0.585 | 0.667 | 7.35E-03 | transcription elongation factor A (SII)-like 8 |
| *Tmeff2* | -0.610 | 0.655 | 2.47E-02 | transmembrane protein with EGF-like and two follistatin-like domains 2 |
| *Tmem100* | -0.969 | 0.511 | 1.29E-04 | transmembrane protein 100 |
| *Tpsb2* | -0.691 | 0.620 | 4.72E-02 | tryptase beta 2 |
| *Ugdh* | -0.575 | 0.672 | 1.23E-02 | UDP-glucose dehydrogenase |
| *Ugt8a* | -0.662 | 0.632 | 6.15E-03 | UDP galactosyltransferase 8A |
| | | | | |
| **Genes involved in adipocytes differentiation** | | | | |
| *Ahnak2* | -0.684 | 0.623 | 2.46E-03 | AHNAK nucleoprotein 2 |
| *Arhgef25* | -0.561 | 0.678 | 2.33E-02 | Rho guanine nucleotide exchange factor (GEF) 25 |
| *Camk2n1* | -0.589 | 0.665 | 4.21E-02 | calcium/calmodulin-dependent protein kinase II inhibitor 1 |
| *Cdkn1c* | -0.592 | 0.663 | 4.51E-03 | cyclin-dependent kinase inhibitor 1C (P57) |
| *Ctsk* | -0.636 | 0.643 | 7.85E-03 | cathepsin K |
| *Ctsl* | -0.434 | 0.740 | 3.01E-02 | cathepsin L |

| | | | | |
|---|---|---|---|---|
| *Eid1* | -0.489 | 0.712 | 3.65E-02 | EP300 interacting inhibitor of differentiation 1 |
| *Ffar2* | -0.624 | 0.649 | 6.28E-03 | free fatty acid receptor 2 |
| *Medag* | -0.615 | 0.653 | 1.13E-02 | mesenteric estrogen dependent adipogenesis |
| *Pros1* | -0.448 | 0.733 | 4.44E-02 | protein S (alpha) |
| *Prrx1* | -0.492 | 0.711 | 3.04E-02 | paired related homeobox 1 |

**Genes involved in inflammation, immune response or expressed in blood cells**

| | | | | |
|---|---|---|---|---|
| *Aif1l* | -0.665 | 0.631 | 1.88E-02 | allograft inflammatory factor 1-like |
| *Akap12* | -0.681 | 0.624 | 2.79E-03 | A kinase (PRKA) anchor protein (gravin) 12 |
| *Alcam* | -0.513 | 0.701 | 1.62E-02 | activated leukocyte cell adhesion molecule |
| *Anxa1* | -0.603 | 0.658 | 1.58E-02 | annexin A1 |
| *Anxa3* | -0.505 | 0.704 | 2.75E-02 | annexin A3 |
| *C1qtnf7* | -0.767 | 0.588 | 5.88E-03 | C1q and tumor necrosis factor related protein 7 |
| *C3ar1* | -0.611 | 0.655 | 2.59E-02 | complement component 3a receptor 1 |
| *Car8* | -0.700 | 0.616 | 6.74E-03 | carbonic anhydrase 8 |
| *Cd209f* | -0.825 | 0.564 | 2.79E-03 | CD209f antigen |
| *Cd248* | -0.839 | 0.559 | 3.42E-04 | CD248 antigen, endosialin |
| *Cd34* | -0.625 | 0.649 | 3.35E-03 | CD34 antigen |
| *Cfh* | -0.825 | 0.565 | 3.42E-04 | complement component factor h |
| *Cma1* | -0.744 | 0.597 | 7.75E-03 | chymase 1, mast cell |
| *Commd1* | -0.559 | 0.679 | 3.57E-02 | COMM domain containing 1 |
| *Cp* | -0.748 | 0.596 | 1.36E-03 | ceruloplasmin |
| *Cpa3* | -0.626 | 0.648 | 3.68E-02 | carboxypeptidase A3, mast cell |
| *Dse* | -0.548 | 0.684 | 1.50E-02 | dermatan sulfate epimerase |
| *Efhd1* | -0.704 | 0.614 | 1.19E-02 | EF hand domain containing 1 |
| *Emilin2* | -0.609 | 0.656 | 2.00E-02 | elastin microfibril interfacer 2 |
| *F13a1* | -0.695 | 0.618 | 4.99E-03 | coagulation factor XIII, A1 subunit |
| *Fcgr3* | -0.626 | 0.648 | 2.01E-02 | Fc receptor, IgG, low affinity III |
| *Fcrls* | -0.786 | 0.580 | 1.13E-02 | Fc receptor-like S, scavenger receptor] |
| *Folr2* | -0.812 | 0.570 | 6.29E-03 | folate receptor 2 (fetal) |
| *Frmd4b* | -0.498 | 0.708 | 3.51E-02 | FERM domain containing 4B] |
| *Hba-a1* | -0.694 | 0.618 | 9.93E-03 | hemoglobin alpha, adult chain 1 |

| | | | | |
|---|---|---|---|---|
| *Il1rl2* | -0.549 | 0.683 | 3.59E-02 | interleukin 1 receptor-like 2 |
| *Mal* | -0.582 | 0.668 | 4.44E-02 | myelin and lymphocyte protein, T cell differentiation protein |
| *Morc4* | -0.619 | 0.651 | 2.11E-02 | microrchidia 4 |
| *Pf4* | -0.730 | 0.603 | 6.34E-03 | platelet factor 4 |
| *Plat* | -0.716 | 0.609 | 2.10E-03 | plasminogen activator, tissue |
| *Ppic* | -0.551 | 0.682 | 1.46E-02 | peptidylprolyl isomerase C |
| *Prnp* | -0.468 | 0.723 | 3.47E-02 | prion protein |
| *Serpinb6a* | -0.684 | 0.622 | 5.76E-04 | serine (or cysteine) peptidase inhibitor, clade B, member 6a |
| *Serpinf1* | -0.556 | 0.680 | 1.43E-02 | serine (or cysteine) peptidase inhibitor, clade F, member 1 |
| *Sulf1* | -0.532 | 0.692 | 1.61E-02 | sulfatase 1 |
| *Wbp5* | -0.683 | 0.623 | 2.34E-04 | WW domain binding protein 5 |
| | | | | |
| **Genes encoding secreted molecules** | | | | |
| *Bmp3* | -0.633 | 0.645 | 4.01E-02 | bone morphogenetic protein 3 |
| *Ccdc80* | -0.849 | 0.555 | 1.09E-04 | coiled-coil domain containing 80 |
| *Chrdl1* | -0.891 | 0.539 | 1.20E-04 | chordin-like 1 |
| *Crispld1* | -0.709 | 0.612 | 6.15E-03 | cysteine-rich secretory protein LCCL domain containing 1 |
| *Fgf2* | -0.576 | 0.671 | 1.56E-02 | fibroblast growth factor 2 |
| *Fstl1* | -0.762 | 0.590 | 1.73E-04 | follistatin-like 1 |
| *Igf1* | -0.710 | 0.611 | 2.04E-04 | insulin-like growth factor 1 |
| *Igf2* | -0.719 | 0.608 | 1.08E-03 | insulin-like growth factor 2 |
| *Igfbp5* | -0.511 | 0.702 | 2.00E-02 | insulin-like growth factor binding protein 5 |
| *Igfbp6* | -0.777 | 0.583 | 5.58E-03 | insulin-like growth factor binding protein 6 |
| *Lep* | -0.507 | 0.704 | 2.18E-02 | leptin |
| *Nucb2* | -0.607 | 0.657 | 5.82E-03 | nucleobindin 2 |
| *Rarres2* | -0.610 | 0.655 | 1.97E-02 | retinoic acid receptor responder (tazarotene induced) 2 |
| *Retnla* | -0.779 | 0.583 | 5.44E-03 | resistin like alpha |
| *Sema3b* | -0.605 | 0.658 | 1.88E-02 | sema domain, immunoglobulin domain (Ig), short basic domain, secreted, (semaphorin) 3B |
| *Sema3d* | -0.677 | 0.625 | 2.55E-03 | sema domain, immunoglobulin domain (Ig), short basic domain, secreted, (semaphorin) 3D |
| *Sfrp2* | -0.796 | 0.576 | 2.10E-03 | secreted frizzled-related protein 2 |
| *Sfrp4* | -1.293 | 0.408 | 3.35E-08 | secreted frizzled-related protein 4 |
| *Smoc1* | -0.542 | 0.687 | 1.18E-02 | SPARC related modular calcium binding 1 |

| | | | | |
|---|---|---|---|---|
| *Sparc* | -0.490 | 0.712 | 2.03E-02 | secreted acidic cysteine rich glycoprotein |
| *Wnt2* | -0.711 | 0.611 | 2.35E-02 | wingless-type MMTV integration site family, member 2 |
| **Genes with unidentified function in the adipose tissue and non coding RNAs** | | | | |
| *AW551984* | -0.645 | 0.640 | 4.93E-02 | expressed sequence AW551984 |
| *Bcas1* | -0.663 | 0.632 | 3.66E-02 | breast carcinoma amplified sequence 1 |
| *C130074G19* | -0.522 | 0.696 | 3.25E-02 | RIKEN cDNA C130074G19 gene |
| *Fam102b* | -0.688 | 0.621 | 1.04E-03 | family with sequence similarity 102, member B] |
| *Fam114a1* | -0.554 | 0.681 | 4.41E-02 | family with sequence similarity 114, member A1 |
| *Fam171b* | -0.759 | 0.591 | 1.58E-02 | family with sequence similarity 171, member B |
| *Gm10093* | -0.809 | 0.571 | 2.36E-02 | histone deacetylase 1 pseudogene |
| *H19* | -0.825 | 0.565 | 2.04E-04 | H19, imprinted maternally expressed transcript (ncRNA) |
| *Snhg18* | -0.615 | 0.653 | 4.61E-02 | small nucleolar RNA host gene 18 (ncRNA) |
| *Zeb2os* | -0.805 | 0.573 | 4.55E-03 | inc finger E-box binding homeobox 2, opposite strand (ncRNA) |
| **Genes with function described in other cell types** | | | | |
| *Gap43* | -0.917 | 0.529 | 1.58E-03 | growth associated protein 43 |
| *Kcnk2* | -0.757 | 0.592 | 2.13E-02 | potassium channel, subfamily K, member 2 |
| *Nbl1* | -0.724 | 0.606 | 1.76E-02 | neuroblastoma, suppression of tumorigenicity 1 |
| *Net1* | -0.781 | 0.582 | 1.42E-03 | neuroepithelial cell transforming gene 1 |
| *Nov* | -0.859 | 0.551 | 1.77E-03 | nephroblastoma overexpressed gene |
| *Nrep* | -0.517 | 0.699 | 3.95E-02 | neuronal regeneration related protein |
| *Ogn* | -0.702 | 0.615 | 2.08E-03 | osteoglycin |
| *Plp1* | -0.579 | 0.669 | 8.66E-03 | proteolipid protein (myelin) 1 |
| *Sbsn* | -0.727 | 0.604 | 3.65E-02 | suprabasin |
| *Sgms2* | -0.650 | 0.637 | 2.34E-02 | sphingomyelin synthase 2 |

| GOTERM_BP_DIRECT | **Down regulated genes in the inguinal subcutaneous adipose tissue of 2-week-old *Egr1*$^{-/-}$ mice** | |
|---|---|---|
| | **Enrichment scores** / P-values | **Genes** |
| **Collagen fibril organization** | **23.8**/ $2.4^{E}$-10 | *Adamts2* a disintegrin-like and metallopeptidase (reprolysin type) with thrombospondin type 1 motif, 2<br>*Col1a1* collagen, type I, alpha 1<br>*Col1a2* collagen, type I, alpha 2<br>*Col3a1* collagen, type III, alpha 1<br>*Col5a2* collagen, type V, alpha 2<br>*Col14a1* collagen, type XIV, alpha 1<br>*Dpt* dermatopontin<br>*Ddr2* discoidin domain receptor family, member 2<br>*Lum* lumican<br>*Sfrp2* secreted frizzled-related protein 2 |
| **Collagen catabolic process** | **21.1**/ $8^{E}$-5 | *Adamts2* a disintegrin-like and metallopeptidase (reprolysin type) with thrombospondin type 1 motif, 2<br>*Ctsk* cathepsin K<br>*Mrc2* mannose receptor, C type 2<br>*Mmp14* matrix metallopeptidase 14<br>*Mmp2* matrix metallopeptidase 2 |
| **Extracellular matrix organization** | **6.7**/ $6.1^{E}$-4 | *Adamtsl4* ADAMTS-like 4<br>*Smoc1* SPARC related modular calcium binding 1<br>*Ccdc80* coiled-coil domain containing 80<br>*Ecm2* extracellular matrix protein 2, female organ and adipocyte specific<br>*Fn1* fibronectin 1<br>*Olfml2b* olfactomedin-like 2B<br>*Postn* periostin |
| **Ossification** | **6.5**/ $2.2^{E}$-3 | *Bmp3* bone morphogenetic protein 3<br>*Chrdl1* chordin-like 1<br>*Ddr2* discoidin domain receptor family, member 2<br>*Igf2* insulin-like growth factor 2 |

| | | |
|---|---|---|
| | | *Lrrc17* leucine rich repeat containing 17<br>*Mmp14* matrix metallopeptidase 14 |
| **Wound healing** | **5.6**/ 3.4$^{E}$-2 | *Col1a1* collagen, type I, alpha 1<br>*Col3a1* collagen, type III, alpha 1<br>*Fgf2* fibroblast growth factor 2<br>*Fn1* fibronectin 1 |
| **Positive regulation of MAPK cascade** | **4.5**/ 2.6$^{E}$-2 | *Igf1* insulin-like growth factor 1<br>*Igf2* insulin-like growth factor 2<br>*Lep* leptin<br>*Lpar1* lysophosphatidic acid receptor 1<br>*Timp2* tissue inhibitor of metalloproteinase 2 |
| **Cell adhesion** | **3.2**/ 1.6$^{E}$-4 | *Cd34* CD34 antigen<br>*Alcam* activated leukocyte cell adhesion molecule<br>*Cdh11* cadherin 11<br>*Col14a1* collagen, type XIV, alpha 1<br>*Dpt* dermatopontin<br>*Emilin2* elastin microfibril interfacer 2<br>*Fap* fibroblast activation protein<br>*Fn1* fibronectin 1<br>*Mfap4* microfibrillar-associated protein 4<br>*Nov* nephroblastoma overexpressed gene<br>*Postn* periostin<br>*Pcdh20* protocadherin 20<br>*Pcdhb14* protocadherin beta 14<br>*Srpx2* sushi-repeat-containing protein, X-linked 2<br>*Thbs3* thrombospondin 3<br>*Vcan* versican |

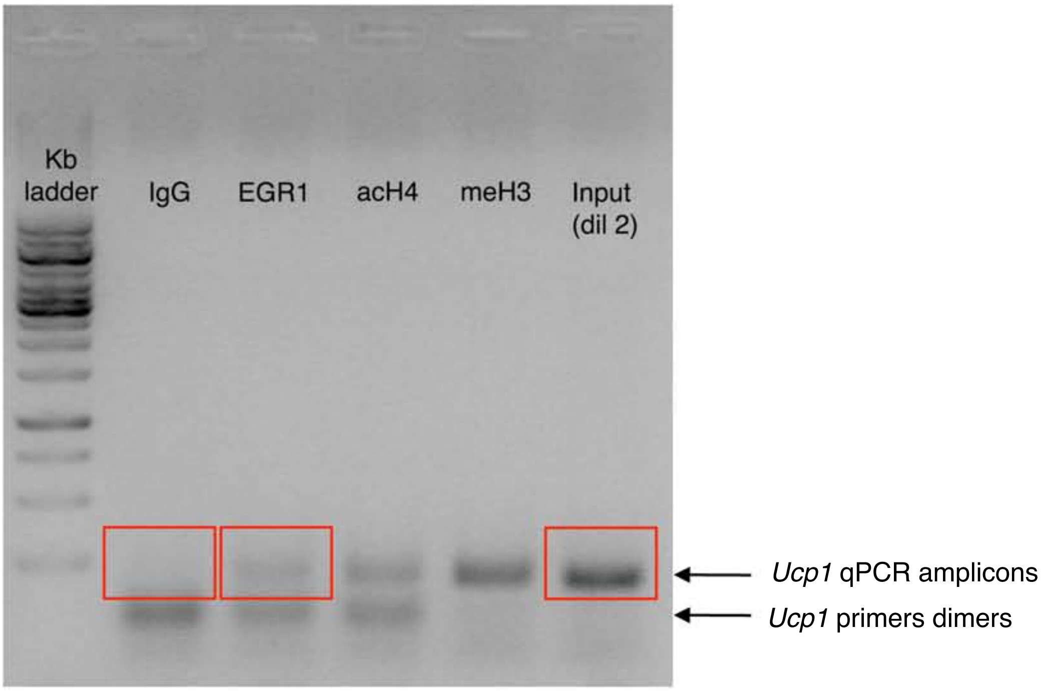
Kb ladder
IgG
EGR1
acH4
meH3
Input (dil 2)
*Ucp1* qPCR amplicons
*Ucp1* primers dimers

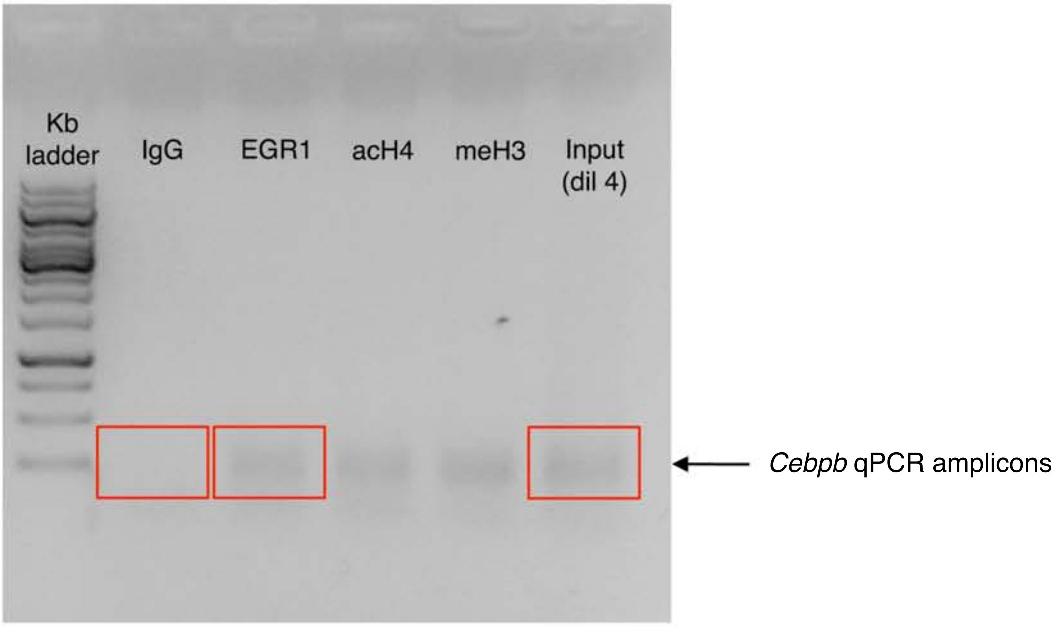
Kb ladder
IgG
EGR1
acH4
meH3
Input (dil 4)
*Cebpb* qPCR amplicons

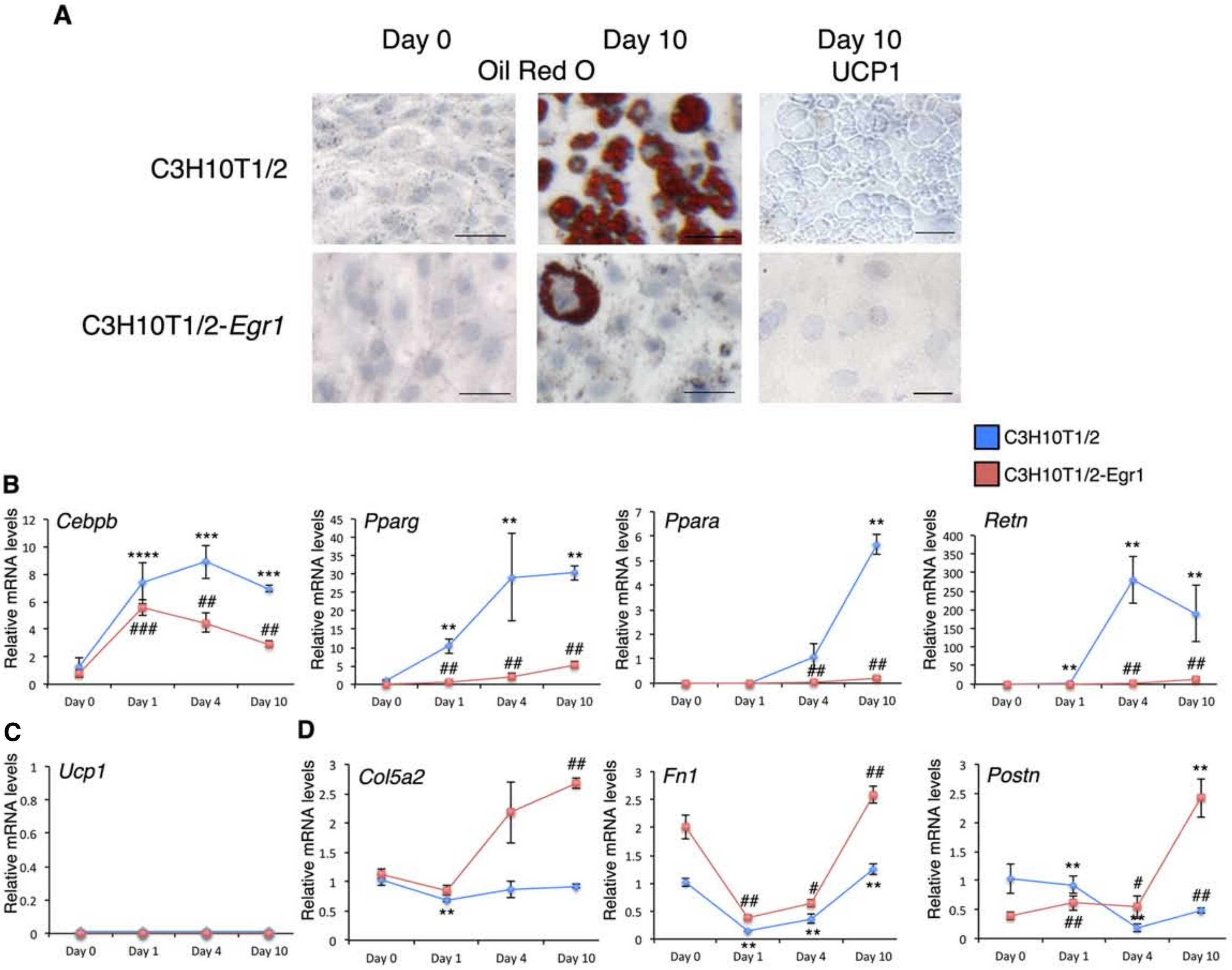
A
Day 0
Day 10
Oil Red O
Day 10
UCP1
C3H10T1/2
C3H10T1/2-Egr1
C3H10T1/2
C3H10T1/2-Egr1
B
Cebpb
Pparg
Ppara
Retn
Relative mRNA levels
Day 0
Day 1
Day 4
Day 10
C
Ucp1
D
Col5a2
Fn1
Postn

| **For RT-qPCR analysis** | | |
|---|---|---|
| **Gene name** | **Forward Primer** | **Reverse Primer** |
| ***Cebpb*** | 5’- CGCCTTTAGACCCATGGAAG | 5’- AGGCAGTCGGGCTCGTAGTAG |
| ***Ppara*** | 5’-AACATCGAGTGTCGAATATGTGG | 5’- CCGAATAGTTCGCCGAAAGAA |
| ***Pparg*** | 5’- TCGGTGATGCACTGCCTATG | 5’- GAGAGGTCCACAGAGCTGATT |
| ***Ppargc1a*** | 5’- TGGACGGAAGCAATTTTTCA | 5’- TTACCTGCGCAAGCTTCTCT |
| ***Dio2*** | 5’- CTTCTCTACCACCACCTTC | 5’- CATCTTCACCCAGTTTAACC |
| ***Pank1*** | 5’- GTTCGCCCAGCATGATTCTC | 5’- CTTAACCAGGGTTCCACCGAT |
| ***Cidea*** | 5’- ACTTCCTCGGCTGTCTCAATGTCA | 5’- TCAGCAGATTCCTTAACACGGCCT |
| ***Ucp1*** | 5’- GGGCATTCAGAGGCAAATCAGCTT | 5’- ACACTGCCACACCTCCAGTCATTA |
| ***Cox8b*** | 5’- AGCCAAAACTCCCACTTCC | 5’- TCTCAGGGATGTGCAACTTC |
| ***Plin5*** | 5’- CAGAGCAAACACCGTACCCAG | 5’- GGGATGGAAAGTAGGGCTAGG |
| ***Ogdh*** | 5’- TATGGCCTACACGAGTCTGAC | 5’- CCAGCCGACGGATGATCTCA |
| ***Sucla2*** | 5’- ACCCTTTCGCTGCATGAATAC | 5’- CTGTGCCTTTATCACAACATCCT |
| ***Col1a1*** | 5’- TGGAGAGAGCATGACCGAT | 5’- GAGCCCTCGCTTCCGTACT |
| ***Col5a2*** | 5’- ACAGGTGAAGTGGGATTCTCA | 5’- CCATAGCACCCATTGGACCA |
| ***Col14a1*** | 5’- TGGAGTATTGGGAGGTTCAACT | 5’- TGCCACTCTATTCTGGGGTCC |
| ***Fn1*** | 5’- CACGTACCTCTTCAAAGTCTTTG | 5’- GGATTGCTTTCCCTGCCCT |

| | | |
|---|---|---|
| ***Postn*** | 5'- TGGTATCAAGGTGCTATCTGCG | 5'- AATGCCCAGCGTGCCATAAA |
| ***Dcn*** | 5'- CTATGTGCCCCTACCGATGC | 5'- CAGAACATGCACCACTCGAAG |
| ***Mmp2*** | 5'- CAAGTTCCCCGGCGATGTC | 5'- TTCTGGTCAAGGTCACCTGTC |
| ***Retn*** | 5'- GCCATCGACAAGAAGATCAA | 5'- CTTCCCTCTGGAGGAGACTG |
| ***ßactin*** | 5'- GATCTGGCACCACACCTTCT | 5'- GGGGTGTTGAAGGTCTCAAA |
| ***Rplp0*** | 5'- ACCTCCTTCTTCCAGGCTTT | 5'- CTCCCACCTTGTCTCCAGTC |
| **For ChIP-qPCR analysis** | | |
| **Promoter name** | **Forward Primer** | **Reverse Primer** |
| ***Cebpb*** | 5'- GAGGGAACTCAGAAGCAAAGT | 5'- AGCCCTCCACCCTATGTAT |
| ***Ppargc1a*** | 5'- GCCTATGAGATCCACGGAAAG | 5'- GTCTCCTTGGCAGTAGAGAATG |
| ***Ucp1*** | 5'- TGTGGCCAGGGCTTTGGGAGT | 5'- AGATTGCCCGGCACTTCTGCG |
| ***Gapdh*** | 5'- CAGGAGCCCAGGGAAGATACAAATA | 5'- ACGCATACACATATACAACCAGTCA |

## Supplementary Figure legends

**Supplementary Figure 1. Cell proliferation in *Egr1*$^{+/+}$ and *Egr1*$^{-/-}$ mice.** SC-WATs of 1-month-old (A,B,C) and 4-months-old (D,E) *Egr1*$^{+/+}$ and *Egr1*$^{-/-}$ mice were sectioned transversely and immuno-stained with KI67 (red). Nuclei were visualized with DAPI (blue). Individual channel or merged channels are indicated in each panel. (**A**) Ki67+ cells were visualized in vessels of SC-WATs of 1-month-old *Egr1*$^{+/+}$ mice. Ki67+ cells were barely observed in SC-WATs of 1 and 4 month *Egr1*$^{+/+}$ mice, as in SC-WATs of 1 and 4-month-old *Egr1*$^{-/-}$ mice. These results indicate that proliferation is not modified in SC-WATs of *Egr1*$^{-/-}$ mice at 1 and 4 months of age compared to control mice. Scale bars in A,B,C and D, 50 $\mu$m.

**Supplementary Figure 2. SC-WAT browning in heterozygous *Egr1*$^{+/-}$ mice.** (**A**) SC-WATs of 4-month-old *Egr1*$^{+/+}$, *Egr1*$^{+/-}$ and *Egr1*$^{-/-}$ mice were sectioned and immuno-stained with UCP1 antibody. Nuclei were visualized with DAPI. Phase, individual or merged channels are indicated in each panels. *Egr1*$^{+/-}$ mice exhibit an intermediate browning of their SC-WAT compared to *Egr1*$^{+/+}$ and *Egr1*$^{-/-}$ mice. (**B**) White and beige adipocyte number was counted in arbitrary unit areas of transverse sections of SC-WAT of 4 month-old Egr1$^{+/+}$ (N=13) and *Egr1*$^{+/-}$ (N=8) mice. Graphs show means of sections for each sample ± standard deviations. Asterisks indicate the p-values obtained using the Mann-Whitney test, comparing beige or white adipocyte number between mutant and control mice * P<0.05, **P<0.01. (**C**) Cell number in SC-WAT in *Egr1*$^{+/+}$ and *Egr1*$^{+/-}$ mice. Number of nuclei (DAPI-positive cells) was counted in arbitrary unit areas of transverse sections of SC-WAT of 4 month-old *Egr1*$^{+/+}$ (N=11) and *Egr1*$^{+/-}$ (N=8) mice. Graphs show means of sections for each sample ± standard deviations. Asterisks indicate the p-values obtained using the Mann-Whitney test, comparing beige or white adipocyte number between mutant and control mice * P<0.05.

**Supplementary Figure 3.** List of upregulated genes in the inguinal subcutaneous adipose tissue of 2-week-old *Egr1*$^{-/-}$ mice versus wild-type mice.

**Supplementary Figure 4.** Gene Ontology analysis of the upregulated genes in the inguinal subcutaneous adipose tissue of *Egr1*$^{+/+}$ versus *Egr1*$^{-/-}$ 2-week-old mice using the DAVID Bioinformatics Resources 6.8.

**Supplementary Figure 5.** List of downregulated genes in the inguinal subcutaneous adipose tissue of 2-week-old *Egr1*$^{-/-}$ mice versus wild-type mice.

**Supplementary Figure 6.** Gene Ontology analysis of downregulated genes in the inguinal subcutaneous adipose tissue of *Egr1*$^{+/+}$ versus *Egr1*$^{-/-}$ 2-week-old mice using the DAVID Bioinformatics Resources 6.8.

**Supplementary Figure 7.** Full scan of the gels used in Figure 3E. Boxed regions correspond to the bands used in the Figure 3E.

**Supplementary Figure 8. *Egr1* gain-of-function decreases white adipose tissue differentiation in mouse mesenchymal stem cells.** (**A**) C3H10T1/2 and C3H10T1/2-*Egr1* cells subjected to white adipocyte differentiation for 10 days were stained with Oil Red O and Hematoxilin/Eosin at Day 0 (confluence) and Day 10, or immuno-stained with UCP1 antibody and counterstained with Hematoxilin/Eosin at Day 10. UCP1 was never expressed in cells cultured in white adipocyte differentiation medium. Scale bars: Oil red O staining 50 $\mu$m, UCP1 immunostaining 25 $\mu$m. (**B-D**) RT-qPCR analysis of the expression levels for the generic adipocyte differentiation genes *Cebpb, Pparag, Ppara*, the white differentiation marker *Retn* (**B**), the thermogenic marker *Ucp1* (**C**), the extracellular matrix genes *Col5a2*, *Fn1* and *Postn* (**D**), in C3H10T1/2 and C3H10T1/2-*Egr1* cells subjected to 10 days of white adipocyte differentiation conditions. *Egr1* repressed the expression of *Cepbb*, *Pparg Ppara* and *Retn*, involved in the white adipocyte differentiation program and activated the expression of ECM genes, *Col5a2*, *Fn1* and *Postn* during white adipocyte differentiation. *Ucp1* expression was not detected in cells cultured in white differentiation conditions. For each time point, graphs show means ± standard deviations of 6 samples. The p values were calculated using the Mann-Withney test. The relative mRNA levels were calculated using the $2^{\wedge-\Delta\Delta Ct}$ method. Asterisks indicate the p-values of gene expression levels in C3H10T1/2-*Egr1* cells or C3H10T1/2 cells compared to Day 0 (*Cebpb*, *Pparg*, *Col5a2*, *Fn1* and *Postn*) or from the first day of gene detection (*Ppara* and *Retn*), **P<0.01, ***P<0,001, ****P<0,0001. # indicate the p-values of gene expression levels in C3H10T1/2-*Egr1* versus C3H10T1/2 cells, for each time point,; # P<0.05, ## P<0.01, ### P<0.001

**Supplementary Table 1.** List of primers used for quantitative PCR.
